# Supplementary material for: In vitro characterisation of the MS2 RNA polymerase complex reveals host factors that modulate emesviral replicase activity
Source: Commun Biol. 2022 Mar 25;5:264. doi: 10.1038/s42003-022-03178-2 (PMC8956599; doi:10.1038/s42003-022-03178-2)
Supplement: Supplementary file 2 — Supplementary Information [file 42003_2022_3178_MOESM2_ESM.pdf]

***In vitro* characterisation of the MS2 RNA polymerase complex reveals host factors that modulate leviviral replicase activity**

Supplementary Information

Alexander Wagner[1], Laura I. Weise[2] and Hannes Mutschler[1*]

[1]Faculty of Chemistry and Chemical Biology, TU Dortmund University,44227 Dortmund, Germany

[2]Max Planck Institute of Biochemistry, 82152 Martinsried, Germany

*To whom correspondence should be addressed. hannes.mutschler@tu-dortmund.de

Table of Contents

Supplementary Materials & Methods: 2

Supplementary Figures: 6

Supplementary Tables: 12

References: 15

SUPPLEMENTARY INFORMATION

Supplementary Materials & Methods:

Chemicals and Oligonucleotides:

All oligos for PCR and cloning, as well as CDSII-T_{24}VN, were purchased from Eurofins Genomics. The template switching oligo TSO-CDSII was purchased from Integrated DNA Technologies (IDT). A 1 mM stock solution of (Z)-4-(3,5-difluoro-4-hydroxybenzylidene)-2-methyl-1-(2,2,2-trifluoroethyl)-1H-imidazol-5(4H)-one (DFHBI-1T, Tocris, 5610) was prepared in dimethylsulfoxide (DMSO, Carl Roth, A994.1). The final 200 µM dilution of DFHBI-1T from this stock was prepared with nuclease-free water.

PCR & IVT for RNA preparation:

The templates for IVTs were synthesized by PCR using the primers specified in Supplementary Table 1, the templates shown in Supplementary Data 4**Error! Reference source not found.** and Q5 Hot Start High-Fidelity 2x Master Mix (NEB), with final concentrations of 0.02 ng / µL for PCR templates and 1 µM for the corresponding primer mix. Annealing temperatures and elongation times are based on standard PCR recommendations by the manufacturer. After the PCR, IVT templates were purified using Monarch® PCR & DNA Cleanup Kit (NEB). For the IVTs, MEGAshortscript™ T7 Transcription Kit (Thermo Scientific Fisher) was used for F30-Bro templates and MEGAscript™ T7 Transcription Kit (Thermo Scientific Fisher) for MS2 templates, following manufacturer's recommendations. After TurboDNAse digest at 37°C for 30 minutes, the final IVT products were purified using Monarch® RNA Cleanup Kit (NEB).

PCR & Assembly for cloning:

In general, plasmids were cloned using NEBuilder® HiFi DNA Assembly Master Mix to combine the pBAD33 backbone with the gene-specific inserts (Supplementary Data 5). Inserts for MS2rep and Qβrep were ordered as gBlocks (IDT). The backbone and other gene-specific inserts were synthesized by PCR with Q5 Hot Start High-Fidelity 2x Master Mix (NEB), using combinations of tag- and gene-specific primers, resulting in an overlap between the different fragments of 30 bp. For HiFi assembly, 100 ng backbone was mixed with one equivalent insert in a 5 µL reaction, incubated at 50°C for 30 minutes and diluted with 15 µL H_2O. Subsequently, 2 µL of each reaction were transformed into chemically competent TOP10 *E. coli* cells (Thermo Fisher Scientific) and plated on LB-Lennox agar containing 34 µL / mL chloramphenicol.

Protein purification:

For protein purification, small cultures of TOP10, or BL21(DE3) *E. coli* cells, respectively, harbouring the expression plasmids were grown at 30°C overnight in LB-Lennox containing 34 µg / mL Chloramphenicol. The overnight cultures were used to inoculate 1 L LB-Lennox with Chloramphenicol and these fresh cultures were grown at 30°C to an OD_{600} of approximately 0.5. Following induction with L-Arabinose for pBAD33 based vectors (Carl Roth) at a final concentration of 0.2% or 1 mM IPTG for pLD1 – 3, respectively, cultures were grown overnight at 16°C. Cells were harvested by centrifugation at 3200 g and 4°C. For lysis, cell pellets were resuspended in 40 mL / L culture HEPES buffer L (50 mM HEPES·KOH pH 7.5, 250 mM NH_4Cl, 10 mM $MgCl_2$, 5 mM DTT, 20 mM imidazole, and 1 mM PMSF) and subsequently lysed by sonication on ice. Cell debris was pelleted by centrifugation at 10,000 g and 4°C for 30 minutes. Before being applied to empty gravity flow columns, the supernatant was incubated at 4°C for 30 minutes with 5 mL / L culture of HisPur™ Ni-NTA resin (Thermo Fisher Scientific) equilibrated with HEPES buffer W (50 mM HEPES·KOH pH 7.5, 250 mM NH_4Cl, 10 mM $MgCl_2$, 5 mM DTT, and 20 mM imidazole). For all LD fractions, washing and elution steps were performed as followed: Washing five times with five column volumes HEPES buffer W, then elution three times with three column volumes HEPES buffer E (HEPES buffer W with 300 mM imidazole). For all individual protein preparations, the resin was washed twice with five column volumes HEPES buffer W, twice with five column volumes HEPES buffer H (HEPES buffer W with 1 M NH_4Cl), and again twice with five column volumes. Elution was performed as described above. Corresponding elution fractions were pooled and concentrated using Merck Millipore Amicon™ Ultra Centrifugal Filter Units (Thermo Fisher Scientific) with a molecular weight cut-off of 10 kDa or 3 kDa for IF1, respectively (3200 g at 4°C). After an initial concentration, the buffer was exchanged to HEPES/glycerine buffer (50 mM HEPES·KOH pH 7.5, 100 mM KCl, 10 mM $MgCl_2$, 7 mM DTT, 30 % glycerol) by diluting the protein concentrate in the spin concentrator as much as possible, then up concentrating again. This process was repeated three times in total, and final protein preparations were frozen in liquid nitrogen and stored at -80°C.

Supplementary real time fluorescence measurements

All experiments for supplementary figures were mixed as described in the method section. Subsequently, they were supplemented as followed:

$[F30\text{-}Bro(+)]_{UTR(+)}$ control: 300 nM MS2rep, 50 nM $[\text{F30-Bro(+)}]_{\text{UTR(+)}}$, 15 µM EF-Ts, and 10 µM DFHBI-1T

Untemplated replication: 200 nM MS2rep or Qβrep, 15 µM EF-Ts, and 1x SYBR™ Green II Nucleic Acid gel stain (Thermo Fisher Scientific)

The reactions measured for inhibition kinetics by IF3 were assembled as described in the method section, to the final concentrations listed in Supplementary Figure 3B. However, for these assays, samples were also overlaid with 20 µL silicon oil (PDMS, viscosity 20 cSt, Sigma-Aldrich) and the fluorescence was measured using a T16-ISO (AXXIN) at 37°C with 12.5 % FAM-LED intensity.

Plaque Assay

To test a possible influence by IF1 and IF3 overexpression on the infectivity of MS2, *F⁺ E. coli* W1485 (ATCC 12435) were transformed with pBAD33-plasmids encoding for IF1, IF3 or AsnRS, respectively, whereas AsnRS served as a control. For each plasmid, one confirmed clone was used to start a 3 mL culture in LB-Lennox with chloramphenicol (34 µg / mL), grown to an OD_{600} 0.5 at 30C°. Subsequently, the expression of the encoded proteins was induced with 0.4 % L-Arabinose and the cells were grown for another 30 minutes at 30°C. Additionally, non-induced cultures were grown in parallel for the non-induced control. The OD_{600} of these cultures were adjusted to 1, by harvesting the cells at 1500 g for 3 minutes and the addition or removal of fresh LB media. For each condition, 100 µL of these cell suspensions were mixed with 10 µL of infectious MS2 dilution (equalling 100 plaque forming units), incubated 30 minutes at room temperature, mixed with 3 mL top-agar (0.6 % agar in LB-Lennox, with Chloramphenicol and 0.4 %-L-Arabinose) and poured onto prewarmed plates containing 1.5 % LB agar. The top agar was allowed to solidify for 15 minutes at room temperature before plates were incubated over night at 37°C.

Fluorescence Anisotropy and Band Shift Assay

The samples for the measurements of fluorescence anisotropy were prepared with $[F30\text{-}Bro(+)]_{UTR(-)}$ (200 nM) and DFHBI-1T (150 nM) as described in the method section for the real time fluorescence measurements. However, in these experiments no MS2rep, EF-Tu, EF-Ts and S1 were added. IF1 or IF3 were added in the following concentrations: 0 µM, 0.12 µM, 0.37 µM, 1.11 µM, 3.33 µM, 10 µM and 30 µM. Fluorescence anisotropy was measured in a Clariostar^{Plus} after incubation for 15 minutes at room temperature after mixing with the following fluorescence settings: λ_{exc} = 482 nm (bandwidth +/- 8 nm) and λ_{em} = 530 nM (bandwidth +/- 20

nm) with dichroic longpass filters (504 nM) in black 384-well plates with clear bottome (Greiner) at 25 °C.

For EMSA experiments, $[F30\text{-}Bro(+)]_{UTR(-)}$ (200 nM), was mixed with IF1/IF3 as described above with the exception that some of the added water was replaced with an appropriate volume of 6.7x loading buffer (0.01 % bromothymol blue, 80 % glycerol, 6.7x SYBR Gold (Thermo Fisher Scientific)) and without supplementation with DFHBI-1T. The initiation factors IF1 and IF3, were supplemented in the following concentrations: 0 µM, 0.56 µM, 0.89 µM, 1.43 µM, 2.29 µM, 3.66 µM, 5.86 µM, 9.38 µM and 15 µM. After incubation at room temperature for 15 minutes, the samples were loaded onto pre-cooled 1.5 % TA agarose gels (40 mM Tris, 20 mM acetic acid), and the gels were run for 3 hours with 1.5 V cm^{-1} in cold TA-buffer. In addition, samples with IF1 were also run on a 1.5 % TB agarose gel with TB-buffer (50 mM Tris, 50 mM boric acid), with identical settings as described above.

Serial Transfer

For serial transfer experiments, reactions were setup by mixing components in the following composition: 15 µM EF-Tu, 15 µM EF-Ts, 1.5 µM S1, 1 µM MS2rep, 0.5 mM of each ATP, GTP, CTP and UTP, 10 mM DTT and 0.5 U / µL RNase inhibitor (moloX). IF1 was supplemented at a final concentration of 15 µM. The final concentration of $MgCl_2$ was 6 mM, with HEPES, KCl and glycerol supplemented in 50 mM, 100 mM and 18 %, respectively. For the first reaction, MS2wt RNA was provided at 50 nM final concentration. Reactions were incubated at 37°C for three hours, frozen in liquid nitrogen, and stored at -80 °C. New reactions were mixed as described above, except that a 1/5 reaction volume (corresponding to MS2wt RNA in H_2O) was replaced with an aliquot from the previous reaction.

Supplementary Figures:

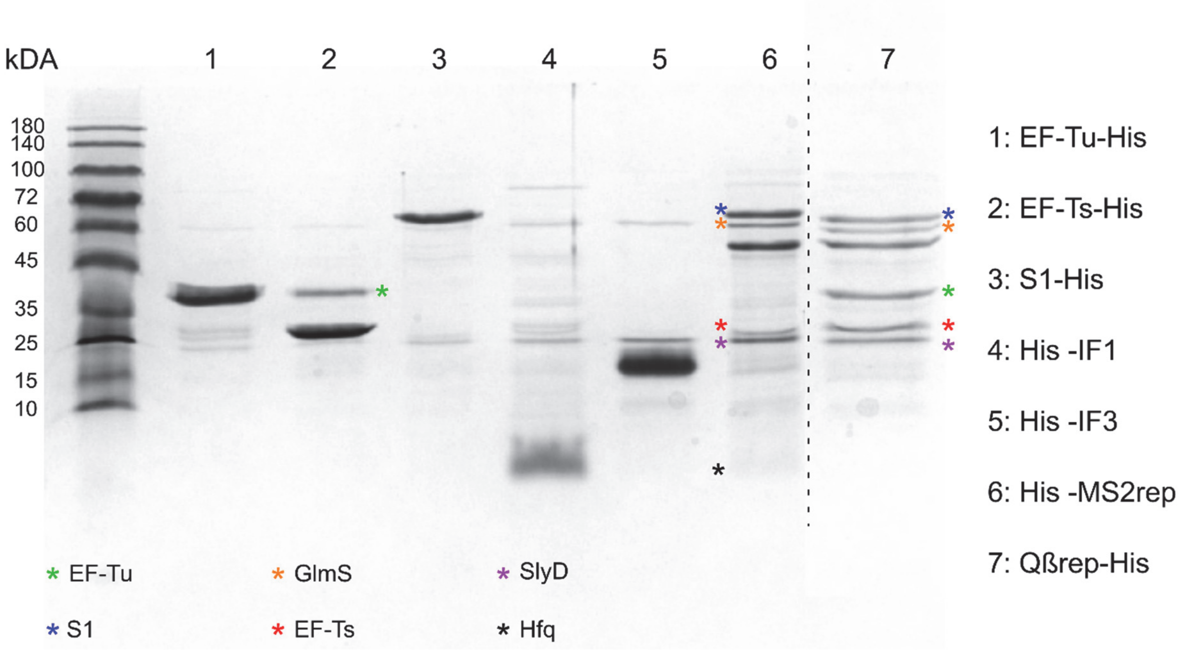

Supplementary Figure 1: Replicases and host factors after affinity-based purification Post-purification 17.5**%** SDS-PAGE of purified cofactors and MS2 or Qβ replicase. 2 µg total protein were loaded per lane. Asterisks indicate co-purified proteins, from top to bottom: EF-Tu (lane 2), S1, GlmS, EF-Ts, SlyD and Hfq (lane 6), S1, GlmS, EF-Tu, EF-Ts, and SlyD (lane 7). For the MS2rep preparation, protein identities of each band were verified by mass spectrometry. SlyD and GlmS are common contaminations during Ni^{2+} IMAC (1). The dotted line indicates where the image was cropped. The ladder used was BlueClassic Prestained Protein Marker (Jena Bioscience).

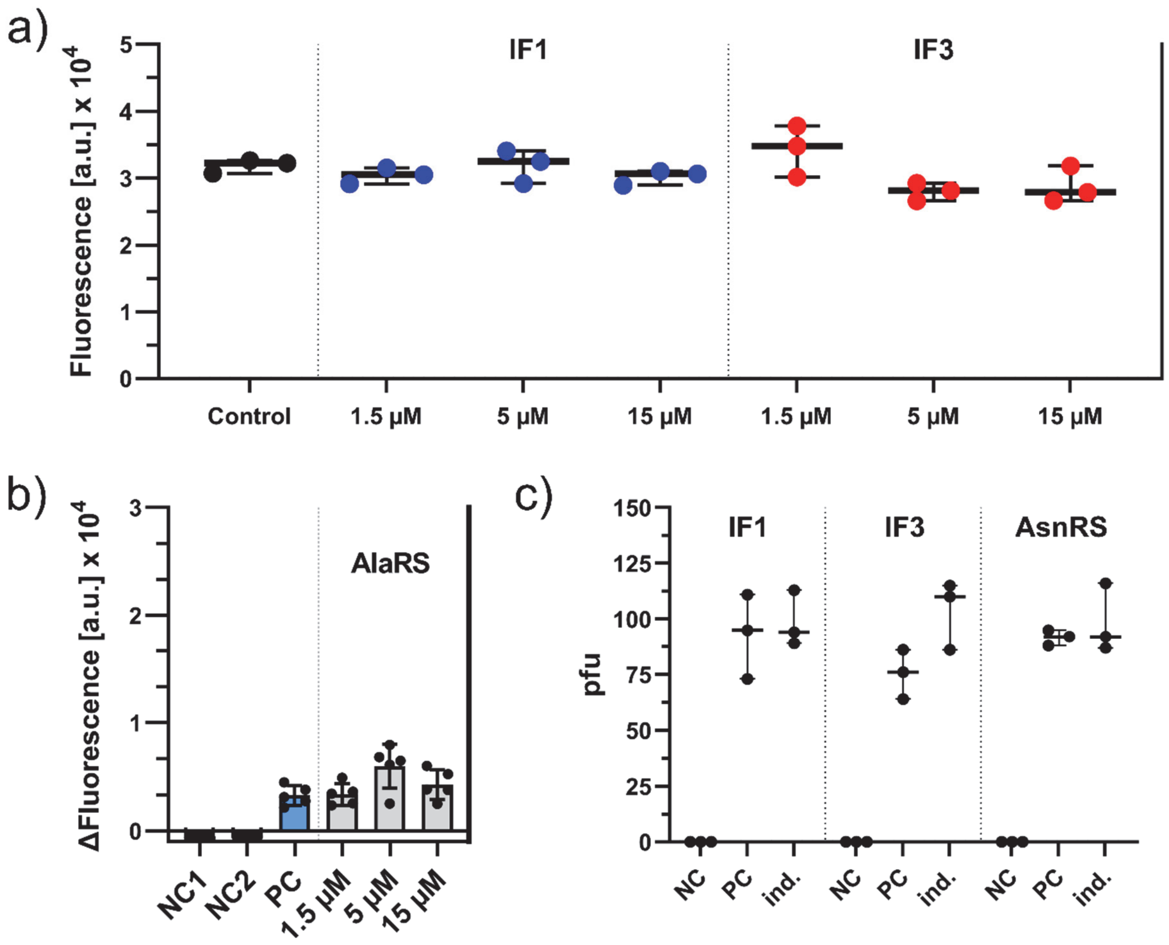

Supplementary Figure 2: Effects of host factors on MS2rep activity and F30-Bro(+) fluorescence. a) IF1 and IF3 have no effect on the fluorescence signal from DFHBI-1T bound F30-Bro(+)]$_{UTR(+)}$. Fluorescence measurements were performed 30 minutes after pre-incubation of the IFs with DFHBI-1T:F30-Bro(+)]$_{UTR(+)}$ complex at 37°C. The control (black) contained 50 nM F30-Bro(+)]$_{UTR(+)}$ RNA, samples with IF1 (blue) or IF3 (red) additionally 1.5 µM, 5 µM or 15 µM of each initiation factor. Error bars indicate standard deviations based on three technical replicates. **b)** Synthesis rate of [F30-Bro(+)]$_{UTR(+)}$ at different AlaRS concentrations. The reactions contained either MS2rep (300 nM), [F30-Bro(-)]$_{UTR(-)}$ (50 nM) as well as EF-Tu (15 µM), EF-Ts (15 µM) and S1 (1.5 µM) for the positive control (PC, blue) and AlaRS as depicted (light grey). In negative controls, samples contained either no MS2rep (NC1, black) or no RNA template (NC2). Error bars indicate standard deviations based on five technical replicates. **c)** A potential *in vivo* effect of IF1 and IF3 overexpression on MS2 replication was analysed through plaque assays. Overexpression of IF1, IF3 or as a control protein (AsnRS) was induced with *L*-arabinose, followed by infection with bacteriophage MS2. Plaque formation was measured for non-infected cultures (NC), infected but not induced cultures (PC) and infected and induced cultures (ind.). Error bars indicate standard deviations from three independent biological replicates

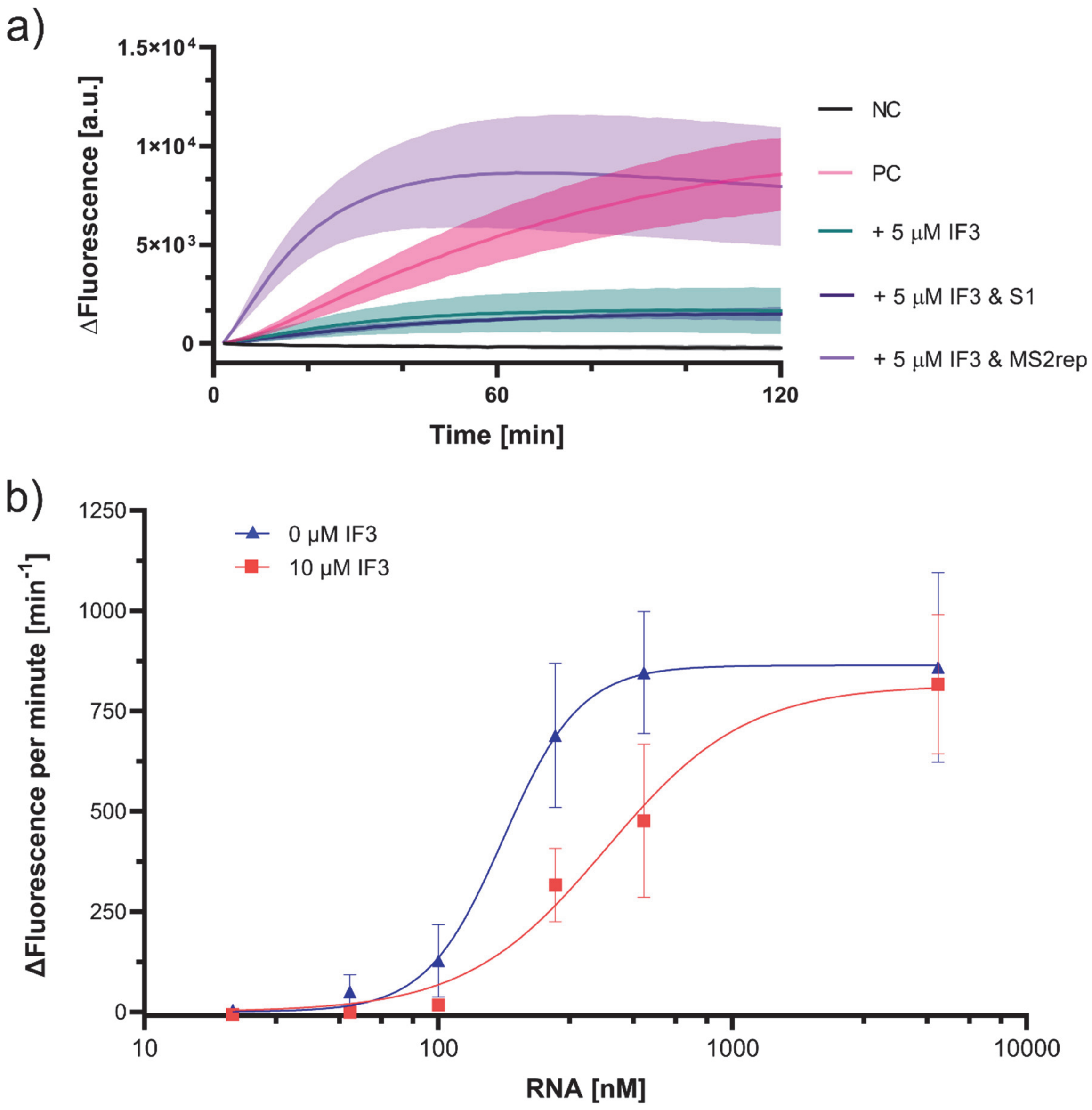

Supplementary Figure 3 Inhibition mode of IF3 on MS2rep activity: a) Influence of IF3 on synthesis of [F30-Bro(+)]$_{UTR(+)}$. Reactions contained 50 nM [F30-Bro(-)]$_{UTR(-)}$ template, 15 µM of each EF-Tu and EF-Ts, 1.5 µM S1, 0.3 µM MS2rep (PC: red), as well the specified combinations of additional protein (5 µM IF3: petrol, 5 µM IF3 and S1: dark purple, 5 µM IF3 and MS2rep: violet). In the negative control (NC: black), MS2rep was omitted. The increase in fluorescence at 37 °C was recorded every 60s. Intervals indicate standard deviations from five technical replicates. **b)** Plot of the initial reaction rates of RNA synthesis by MS2rep without (blue, triangle) or in presence of 10 µM IF3 (red, square) as a function of [F30-Bro(-)]$_{UTR(-)}$ template concentration. The substrate RNA was supplemented in the following concentrations: 20 nM, 50 nM, 100 nM, 250 nM, 500 nM and 5000 nM. Reaction rates were derived from real time fluorescence measurements using a linear fit for the initial linear phase of RNA synthesis. Bars represent standard deviations from three independent technical replicates. Binding isotherms were fit using the Hill equation $Y=v_{max}*X^n/(K^n + X^n)$, where X is the RNA template concentration and K the apparent half-saturation concentration of the maximal rate of the fluorescence increase, and v_{max} the maximum apparent rate of the fluorescence increase at saturating substrate concentration at the given enzyme concentration.

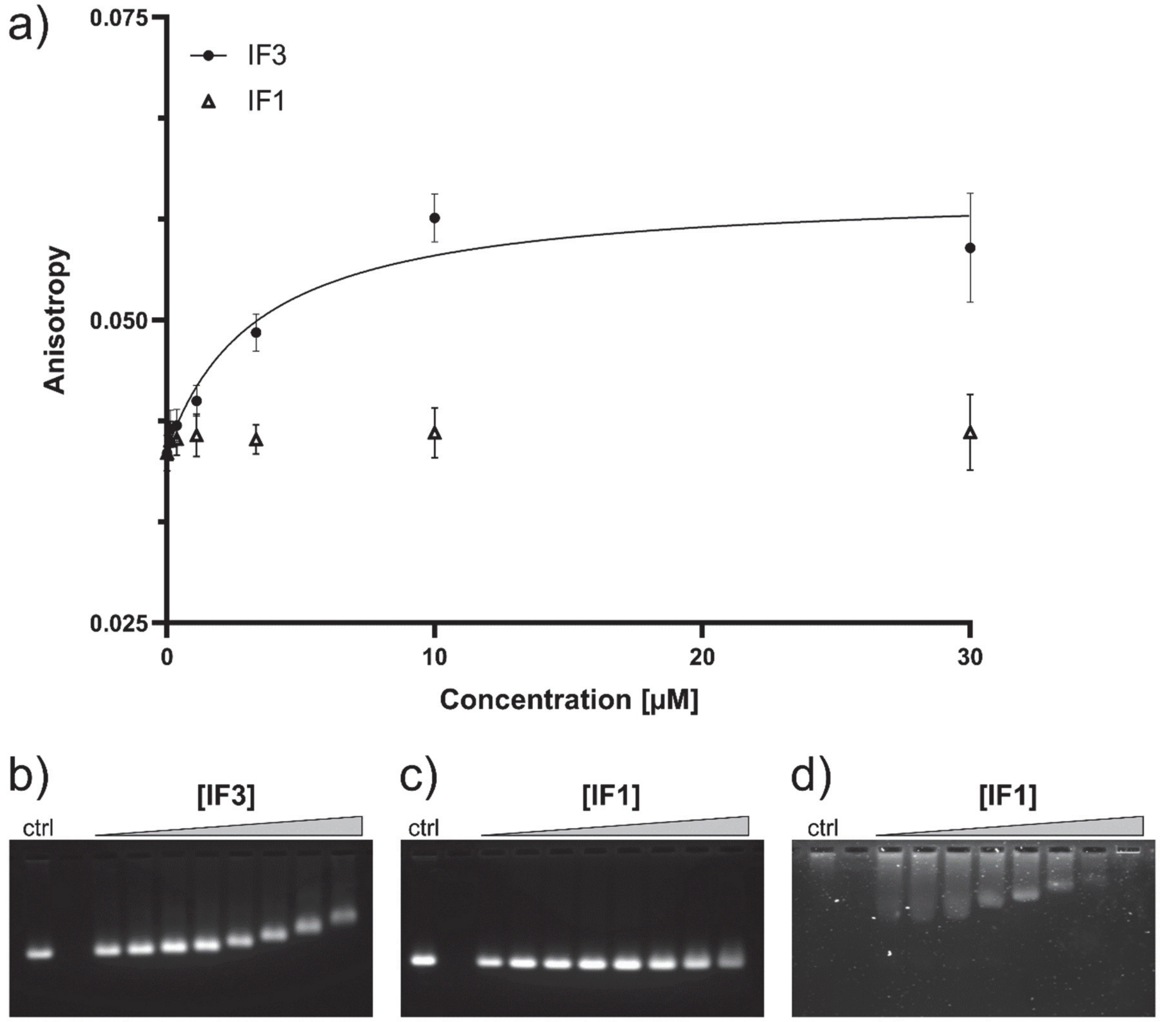

Supplementary Figure 4: Binding of initiation factors 1 and 3 to $[\text{F30-Bro(+/-)}]_{UTR(-)}$ template RNA a) Fluorescence anisotropy of DFHBI-1T bound to $[\text{F30-Bro(+)}]_{UTR(-)}$ (200 nM), plotted against increasing concertation of IF1 (triangled) and IF3 (circles). IF1 and IF3 were added in following concentrations: 0 µM, 0.12 µM, 0.37 µM, 1.11 µM, 3.33 µM, 10 µM and 30 µM. Error bars report standard deviation derived from three independent technical replicates. Data points obtained for IF3 were fitted using an equation for specific binding on one site with offset ($Y=r_{max}*X/(K_D + X)+r_0$, where X is the concentration of IF3 and K_D the apparent dissociation constant. **b) - d)** Gel electrophoresis of the band shift samples for IF3 on a 1.5 % TA agarose gel (**b)**) and IF1 on a 1.5 % TA agarose gel (**c)**) or IF1 on a 1.5 % TB agarose gel (**d)**), respectively. The leftmost band (ctrl, $[\text{F30-Bro(-)}]_{UTR(-)}$) serves as a reference for unbound RNA. IF1 and IF3 were supplemented in following concentrations: 0.56 µM, 0.89 µM, 1.43 µM, 2.29 µM, 3.66 µM, 5.86 µM, 9.38 µM and 15 µM.

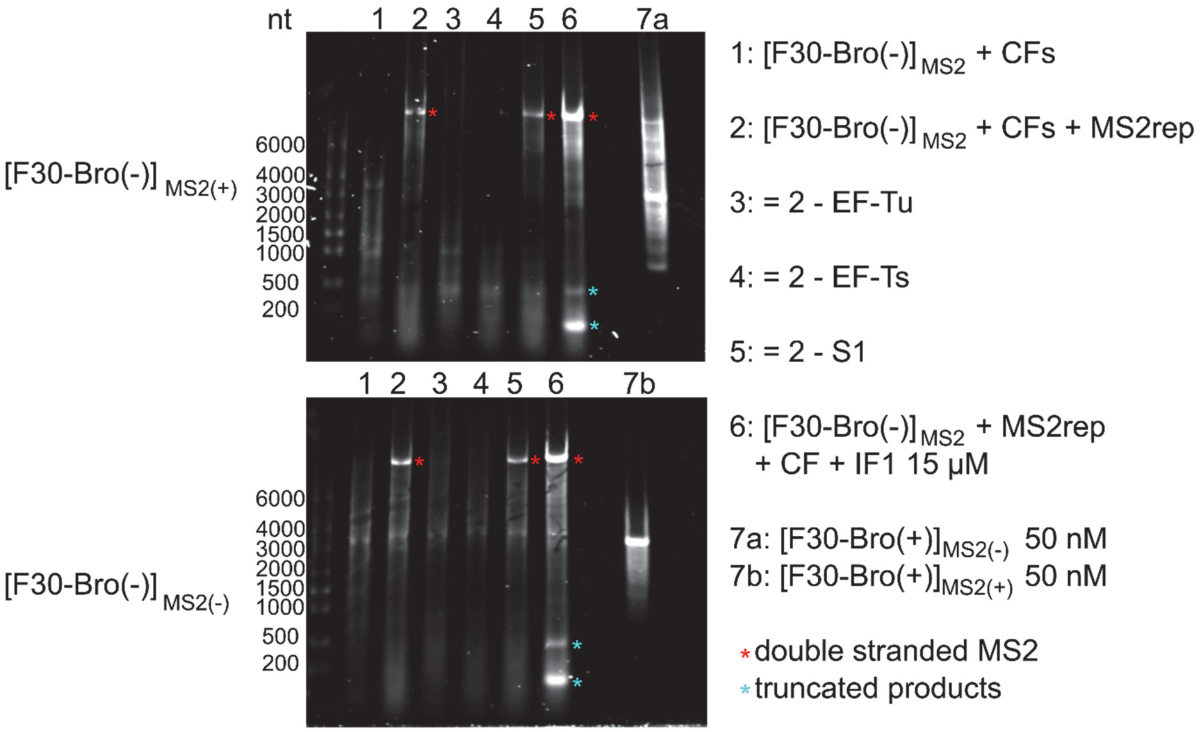

Supplementary Figure 5: Replication of full length MS2-Bro RNA. Gel electrophoresis (1 % agarose) of samples from the replication of full-length MS2 RNA constructs [F30-Bro(-)]$_{MS2(-)}$ and [F30-Bro(-)]$_{MS2(+)}$, taken after incubation at 37°C for six hours. Core factors (CF) were EF-Ts (15 µM), EF-Tu (15 µM), and S1 (1.5 µM). Red asterisks indicate double-stranded MS2 RNA that migrates at approximately 7200 nt. Purple asterisks indicate small, putatively replicative RNA species. Reactions were prepared as described in the method section using either (+) or (-) strand as templates as indicated on the left side, and by either adding or omitting host factors, as indicated on the right side. The samples in lane 7 serve as a size and quantity standard for the expected single stranded product RNA. The ladder used was RiboRuler High Range RNA Ladder (Thermo Fisher Scientific).

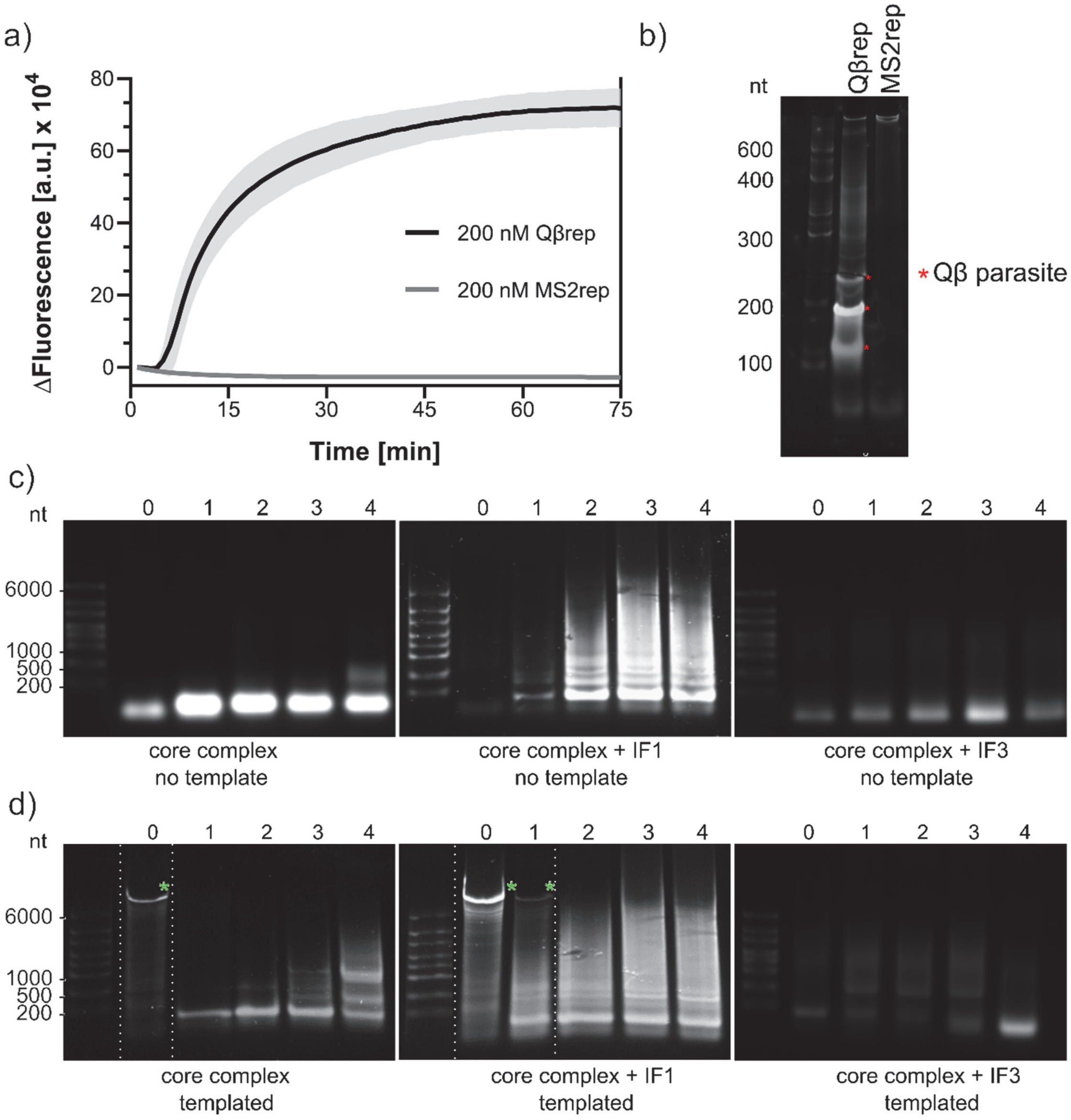

Supplementary Figure 6: Emergence of parasitic RNAs a) Fluorescence change over time for untemplated reactions with Qβ replicase (black) or MS2rep (grey), incubated at 37°C for 75 minutes. Error bars are based on standard deviation from three independent technical replicates. **b)** Gel electrophoresis (10% TBU PAGE) of samples taken from the reactions shown in a). Asterisks indicate putative small self-replicating RNA species. RiboRuler Low Range RNA Ladder (Thermo Fisher Scientific) was used as a molecular weight standard. **c – d**) Gel electrophoresis (1 % agarose) of serial transfer samples, taken after 3 hours of incubation at 37°C. Numbers above the lanes indicate the number of transfers, the asterisks indicate double stranded MS2 RNA. All reactions contained 15 µM EF-Tu and EF-Ts, as well as 1.5 µM S1. For c) **Left:** MS2rep core complex (1 µM) alone. **Middle:** MS2rep core complex (1 µM) with IF1 (15 µM). **Right:** MS2rep core complex (1 µM) with IF3 (15 µM). For d) Identical setup of reaction conditions, however, each of the initial reaction mixes was additionally programmed with 50 nM MS2(+) RNA. The molecular weight standard used in this experiment was RiboRuler High Range RNA Ladder (Thermo Fisher Scientific). White dotted lines indicate, where separated lanes from the same gel were positioned next to each other for improved visuatisation

Supplementary Tables

***Supplementary Table 1**: Primer sequences*

**Primer Name**	**Sequence**	**T_a**
PCR for [F30-Bro]$_{UTR/MS2(+)}$ *and MS2wt(+)*		
T7_MS2(+)aP_fw	GAAATAATACGACTCACTATAGGGTGGGACCCCTTTC	67°C
MS2_3endlong_rev	TGGGTGGTAACTAGCCAAGCAGC	
PCR for [F30-Bro]$_{UTR/MS2(-)}$		
T7_MS2(-)aP_fw	TAATACGACTCACTATAGGGTGGTAAC	66°C
MS2(-)aP_rv	GGGTGGGACCCCTTTC	
RT & Template Switching		
CDSII-T_{24}VN	AAGCAGTGGTATCAACGCAGAGTTTTTTTTTTTTTTTTTTTTTTTTVN	-
TSO-CDSII	GCTAATAAGCAGTGGTATCAACGCAGAGGCAGAGTACAT rGrGrG	-

Supplementary Table 2: Composition of in-house PURE

**Component**	**Stock**	**Final**
**LD1 (10 x)**		
RF1	10 x	1 x
HisRS	10 x	1 x
TyrRS	10 x	1 x
CysRS	10 x	1 x
TrpRS	10 x	1 x
SerRS	10 x	1 x
ValRS	10 x	1 x
MetRS	10 x	1 x
ArgRS	10 x	1 x

GlnRS	10 x	1 x
LeuRS	10 x	1 x
ThrRS	10 x	1 x
LysRS	10 x	1 x
**LD2 (5 x), reconstituted**		
AsnRS	26 µM	5.2 µM
IleRS	28 µM	5.6 µM
AlaRS	31.25 µM	6.25 µM
PheRS1	50.5 µM	10.1 µM
PheRS2	50.5 µM	10.1 µM
MTF	52.5 µM	10.5 µM
EF-Ts	75 µM	15 µM
IF3	25 µM	5 µM
IF1	84 µM	16.8 µM
**LD3 (10 x)**		
AspRS	10 x	1 x
ProRS	10 x	1 x
GlyRS1	10 x	1 x
GlyRS2	10 x	1 x
GluRS	10 x	1 x
RF3	10 x	1 x
RF2	10 x	1 x
RRF	10 x	1 x
EF-G	10 x	1 x
IF2	10 x	1 x
**Enzyme mix (6 x)**		
T7 RNAP	120 ng/µL	20 ng/µL

Myokinase	30 ng/µL	5 ng/µL
Creatine phosphokinase	60 ng/µL	10 ng/µL
Nucleoside-diphosphate kinase	12 ng/µL	2 ng/µL
RNAse Inhibitor	1.5 U/µL	0.25 U/µL
Inorganic pyrophosphatase	6 U/µL	1 U/µL
EF-Tu	30 µM	5 µM
Ribosomes	10.8 µM	1.8 µM
HEPES·KOH pH 8.0	50 mM	8.3 mM
DTT	6 mM	1 mM
Glycerol	2 %	0.3 %
**Energy mix (4 x)**		
Potassium Glutamate	400 mM	100 mM
Spermidine	10mM	2.5 mM
ATP	8 mM	2 mM
GTP	8 mM	2 mM
CTP	4 mM	1 mM
UTP	4 mM	1 mM
Sodium Creatine Phosphate	80 mM	20 mM
Folinic Acid	40 mM	10 mM
HEPES-KOH pH 7.5	200 mM	50 mM
Mg(OAc)2	52 mM	13 mM
DTT	20 mM	5 mM
tRNA	OD_{260} 216	OD_{260} 54
Amino Acid mix	4 mM	1 mM

Supplementary Table 3*: Sequence of MSRP-22 sense strand*

**MSRP-22**
UGGGUGGGACCCCUUUCGGGGUCCUGCUCAACUUCCUGUCGAGCUAAUGCCAUU<br>UUUAAUGUCUUUAGCGAGACGCUACCAUGGCUAUCGCUGUAGGUAGCCGGAAUUC<br>CAUUCCUAGGGCUCCACCGAAAGGUGGGCGGGGCUUCGGCCCAGGGACCUCCCC<br>CUAAAGAGAGGACCCGGGAUUCUACCGGUUUGGUAACUAGCUGAUUGGCUAGUUA<br>CCACCCC

Supplementary References:

1. Bolanos-Garcia,V.M. and Davies,O.R. (2006) Structural analysis and classification of native proteins from E. coli commonly co-purified by immobilised metal affinity chromatography. *Biochim. Biophys. Acta - Gen. Subj.*, **1760**, 1304–1313.
