# Supplementary material for: In vitro characterisation of the MS2 RNA polymerase complex reveals host factors that modulate emesviral replicase activity
Source: Commun Biol. 2022 Mar 25;5:264. doi: 10.1038/s42003-022-03178-2 (PMC8956599; doi:10.1038/s42003-022-03178-2)
Supplement: Supplementary file 3 — Description of Additional Supplementary Files [file 42003_2022_3178_MOESM3_ESM.pdf]

Description of Additional Supplementary Files

File name: Supplementary Data 1
Description: All mass spectrometry data used for protein identification of co-purified proteins in the MS2rep subunit preparation.

File name: Supplementary Data 2
Description: Source data for all figures and supplementary figures prepared and analysed as part of this study.

File name: Supplementary Data 3
Description: Sequencing chromatogram for MSRP-22 cDNA.

File name: Supplementary Data 4
Description: All DNA templates used for IVT.

File name: Supplementary Data 5
Description: Sequences of all plasmids used in this study.
