# Supplementary material for: In vitro characterisation of the MS2 RNA polymerase complex reveals host factors that modulate emesviral replicase activity
Source: Commun Biol. 2022 Mar 25;5:264. doi: 10.1038/s42003-022-03178-2 (PMC8956599; doi:10.1038/s42003-022-03178-2)
Supplement: Supplementary file 7 — Supplementary Data 4 [file 42003_2022_3178_MOESM7_ESM.docx]

| **[F30-Bro(-)]_UTR(-)_** |
| --- |
| T7 promoter **Broccoli core (-)**  TAATACGACTCACTATAGGGTGGTAACTAGCCAAGCAGCTAGTTACCAAATCGGGAGAATCCCGGGTCCTCTCTTTAGGGGGAGGTCCCTGGGCCGAAGCCCGCCCACCTTTCGGTGGAGCCGGACCGCTTTCGCACCCGTGCTCTTTCGAGCACACCCACCCCGTTTACGGGGGTCCCTCGGTCAGCTACCGAGGAGTTGCCATGAATGATCCCGAAGGATCATCAGAGTATGTGGG**AGCCCACACTCTACTCGACAGATACGAATATCTGGACCCGACCGTCT**CCCACATACACATGGCAAAAACCTCCTAGGAATGGAATTCCGGCTACCTACAGCGATAGCCATGGTAGCGTCTCGCTAAAGACATTAAAAATGGCATTAGCTCGACAGGAAGTTGAGCAGGACCCCGAAAGGGGTCCCACCC |
| **[F30-Bro(+)]_UTR(+)_** |
| T7 promoter **Broccoli core (+)**  TAATACGACTCACTATAGGGTGGGACCCCTTTCGGGGTCCTGCTCAACTTCCTGTCGAGCTAATGCCATTTTTAATGTCTTTAGCGAGACGCTACCATGGCTATCGCTGTAGGTAGCCGGAATTCCATTCCTAGGAGGTTTTTGCCATGTGTATGTGGG**AGACGGTCGGGTCCAGATATTCGTATCTGTCGAGTAGAGTGTGGGCT**CCCACATACTCTGATGATCCTTCGGGATCATTCATGGCAACTCCTCGGTAGCTGACCGAGGGACCCCCGTAAACGGGGTGGGTGTGCTCGAAAGAGCACGGGTGCGAAAGCGGTCCGGCTCCACCGAAAGGTGGGCGGGCTTCGGCCCAGGGACCTCCCCCTAAAGAGAGGACCCGGGATTCTCCCGATTTGGTAACTAGCTGCTTGGCTAGTTACCACCC |
| **[F30-Bro(+)]_UTR(-)_** |
| T7 promoter **Broccoli core (+)**  TAATACGACTCACTATAGGGTGGTAACTAGCCAAGCAGCTAGTTACCAAATCGGGAGAATCCCGGGTCCTCTCTTTAGGGGGAGGTCCCTGGGCCGAAGCCCGCCCACCTTTCGGTGGAGCCGGACCGCTTTCGCACCCGTGCTCTTTCGAGCACACCCACCCCGTTTACGGGGGTCCCTCGGTCAGCTACCGAGGAGTTGCCATGTGTATGTGGG**AGACGGTCGGGTCCAGATATTCGTATCTGTCGAGTAGAGTGTGGGCT**CCCACATACTCTGATGATCCTTCGGGATCATTCATGGCAAAAACCTCCTAGGAATGGAATTCCGGCTACCTACAGCGATAGCCATGGTAGCGTCTCGCTAAAGACATTAAAAATGGCATTAGCTCGACAGGAAGTTGAGCAGGACCCCGAAAGGGGTCCCACCC |
| **[F30-Bro(-)]_UTR(+)_** |
| T7 promoter **Broccoli core (-)**  TAATACGACTCACTATAGGGTGGGACCCCTTTCGGGGTCCTGCTCAACTTCCTGTCGAGCTAATGCCATTTTTAATGTCTTTAGCGAGACGCTACCATGGCTATCGCTGTAGGTAGCCGGAATTCCATTCCTAGGAGGTTTTTGCCATGAATGATCCCGAAGGATCATCAGAGTATGTGGG**AGCCCACACTCTACTCGACAGATACGAATATCTGGACCCGACCGTCT**CCCACATACACATGGCAACTCCTCGGTAGCTGACCGAGGGACCCCCGTAAACGGGGTGGGTGTGCTCGAAAGAGCACGGGTGCGAAAGCGGTCCGGCTCCACCGAAAGGTGGGCGGGCTTCGGCCCAGGGACCTCCCCCTAAAGAGAGGACCCGGGATTCTCCCGATTTGGTAACTAGCTGCTTGGCTAGTTACCACCC |
| **[F30-Bro(-)]_MS2(+)_** |
| T7 promoter **Broccoli core (-)**  TAATACGACTCACTATAGGGTGGGACCCCTTTCGGGGTCCTGCTCAACTTCCTGTCGAGCTAATGCCATTTTTAATGTCTTTAGCGAGACGCTACCATGGCTATCGCTGTAGGTAGCCGGAATTCCATTCCTAGGAGGTTTGACCTGTGCGAGCTTTTAGTACCCTTGATAGGGAGAACGAGACCTTCGTCCCCTCCGTTCGCGTTTACGCGGACGGTGAGACTGAAGATAACTCATTCTCTTTAAAATATCGTTCGAACTGGACTCCCGGTCGTTTTAACTCGACTGGGGCCAAAACGAAACAGTGGCACTACCCCTCTCCGTATTCACGGGGGGCGTTAAGTGTCACATCGATAGATCAAGGTGCCTACAAGCGAAGTGGGTCATCGTGGGGTCGCCCGTACGAtGAGAAAGCCGGTTTCGGCTTCTCCCTCGACGCACGCTCCTGCTACAGCCTCTTCCCTGTAAGCCAAAACTTGACTTACATCGAAGTGCCGCAGAACGTTGCGAACCGGGCGTCGACCGAAGTCCTGCAAAAGGTCACCCAGGGTAGTTTTAACCTTGGTGTTGCTCTAGCAGAGGCCAGGTCGACAGCCTCACAACTCGCGACGCAAACCATTGCGCTCGTGAAGGCGTACACTGCCGCTCGTCGCGGTAATTGGCGCCAGGCGCTCCGCTACCTTGCCCTAAACGAAGATCGAAAGTTTCGATCAAAACACGTGGCCGGCAGGTGGTTGGAGTTGCAGTTCGGTTGGTTACCACTAATGAGTGATATCCAGGGTGCATATGAGATGCTTACGAAGGTTCACCTTCAAGAGTTTCTTCCTATGAGAGCCGTACGTCAGGTCGGTACTAACATCAAGTTAGATGGCCGTCTGTCGTATCCAGCTGCAAACTTCCAGACAACGTGCAACATATCGCGACGTATCGTGATATGGTTTTACATAAACGATGCACGTTTGGCATGGTTGTCGTCTCTAGGTATCTTGAACCCACTAGGTATAGTGTGGGAAAAGGTGCCTTTCTCATTCGTTGTCGACTGGCTCCTACCTGTAGGTAACATGCTCGAGGGCCTTACGGCCCCCGTGGGATGCTCCTACATGTCAGGAACAGTTACTGACGTAATAACGGGTGAGTCCATCATAAGCGTTGACGCTCCCTACGGGTGGACTGTGGAGAGACAGGGCACTGCTAAGGCCCAAATCTCAGCCATGCATCGAGGGGTACAATCCGTATGGCCAACAACTGGCGCGTACGTAAAGTCTCCTTTCTCGATGGTCCATACCTTAGATGCGTTAGCATTAATCAGGCAACGGCTCTCTAGATAG**AGCCCACACTCTACTCGACAGATACGAATATCTGGACCCGACCGTCT**CTATCTAGAGGGCCCTCAACCGGAGTTTGAAGCATGGCTTCTAACTTTACTCAGTTCGTTCTCGTCGACAATGGCGGAACTGGCGACGTGACTGTCGCCCCAAGCAACTTCGCTAACGGGGTCGCTGAATGGATCAGCTCTAACTCGCGTTCACAGGCTTACAAAGTAACCTGTAGCGTTCGTCAGAGCTCTGCGCAGAATCGCAAATACACCATCAAAGTCGAGGTGCCTAAAGTGGCAACCCAGACTGTTGGTGGTGTAGAGCTTCCTGTAGCCGCATGGCGTTCGTACTTAAATATGGAACTAACCATTCCAATTTTCGCTACGAATTCCGACTGCGAGCTTATTGTTAAGGCAATGCAAGGTCTCCTAAAAGATGGAAACCCGATTCCCTCAGCAATCGCAGCAAACTCCGGCATCTACTAATAGACGCCGGCCATTCAAACATGAGGATTACCCATGTCGAAGACAACAAAGAAGTTCAACTCTTTATGTATTGATCTTCCTCGCGATCTTTCTCTCGAAATTTACCAATCAATTGCTTCTGTCGCTACTGGAAGCGGTGATCCGCACAGTGACGACTTTACAGCAATTGCTTACTTAAGGGACGAATTGCTCACAAAGCATCCGACCTCAGGTTCCGGTAATGACGAGGCGACCCGTCGTACCTTAGCTATCGCTAAGCTACGGGAGGCGAATGATCGGTGCGGTCAGATAAATAGAGAAGGTTTCTTACATGACAAATCCTTGTCATGGGATCCGGATGTTTTACAAACCAGCATCCGTAGCCTTATTGGCAACCTCCTCTCTGGCTACCGATCGTCGTTGTTTGGGCAATGCACGTTCTCCAACGGTGCctCTATGGGGCACAAGTTGCAGGATGCAGCGCCTTACAAGAAGTTCGCTGAACAAGCAACCGTTACCCCCCGCGCTCTGAGAGCGGCTCTATTGGTCCGAGACCAATGTGCGCCGTGGATCAGACACGCGGTCCGCTATAACGAGTCATATGAATTTAGGCTCGTTGTAGGGAACGGAGTGTTTACAGTTCCGAAGAATAATAAAATAGATCGGGCTGCCTGTAAGGAGCCTGATATGAATATGTACCTCCAGAAAGGGGTCGGTGCCTTtATCAGACGCCGGCTCAAATCCGTTGGTATAGACCTAAATGATCAATCGATCAACCAGCGTCTGGCTCAGCAGGGCAGCGTAGATGGTTCGCTTGCGACGATAGACTTATCGTCTGCATCCGATTCCATCTCCGATCGCCTGGTGTGGAGTTTTCTCCCACCTGAGCTATATTCATATCTCGATCGTATCCGCTCACACTACGGAATCGTAGATGGCGAGACGATACGATGGGAACTATTTTCCACAATGGGAAATGGGTTCACATTTGAGCTAGAGTCCATGATATTCTGGGCAATAGTCAAAGCGACCCAAATCCATTTTGGTAACGCCGGAACCATAGGCATCTACGGGGACGATATTATATGTCCCAGTGAGATTGCACCCCGTGTGCTAGAGGCACTTGCCTACTACGGTTTTAAACCGAATCTTCGTAAAACGTTCGTGTCCGGGCTCTTTCGCGAGAGCTGCGGCGCGCACTTTTACCGTGGTGTCGATGTCAAACCGTTTTACATCAAGAAACCTGTTGACAATCTCTTCGCCCTGATGCTGATATTAAATCGGCTACGGGGTTGGGGAGTTGTCGGAGGTATGTCAGATCCACGCCTCTACAAGGTGTGGGTACGGCTCTCCTCCCAGGTGCCTTCGATGTTCTTCGGTGGGACGGACCTCGCTGCCGACTACTACGTAGTCAGCCCGCCTACGGCAGTCTCGGTATACACCAAGACTCCGCACGGGCGGCTGCTCGCGGATACCCGTACCTCGGGTTTCCGTCTTGCTCGTATCGCTCGAGAACGCAAGTTCTTCAGCGAAAAGCACGACAGTGGTCGCTACATAGCGTGGTTCCATACTGGAGGTGAAATCACCGACAGCATGAAGTCCGCCGGCGTGCGCGTTATACGCACTTCGGAGTGGCTAACGCCGGTTCCCACATTCCCTCAGGAGTGTGGGCCAGCGAGCTCTCCTCGGTAGCTGACCGAGGGACCCCCGTAAACGGGGTGGGTGTGCTCGAAAGAGCACGGGTCCGCGAAAGCGGTGGCTCCACCGAAAGGTGGGCGGGCTTCGGCCCAGGGACCTCCCCCTAAAGAGAGGACCCGGGATTCTCCCGATTTGGTAACTAGCTGCTTGGCTAGTTACCACCC |
| **[F30-Bro(+)]_MS2(-)_** |
| T7 promoter **Broccoli core (+)**  TAATACGACTCACTATAGGGTGGTAACTAGCCAAGCAGCTAGTTACCAAATCGGGAGAATCCCGGGTCCTCTCTTTAGGGGGAGGTCCCTGGGCCGAAGCCCGCCCACCTTTCGGTGGAGCCACCGCTTTCGCGGACCCGTGCTCTTTCGAGCACACCCACCCCGTTTACGGGGGTCCCTCGGTCAGCTACCGAGGAGAGCTCGCTGGCCCACACTCCTGAGGGAATGTGGGAACCGGCGTTAGCCACTCCGAAGTGCGTATAACGCGCACGCCGGCGGACTTCATGCTGTCGGTGATTTCACCTCCAGTATGGAACCACGCTATGTAGCGACCACTGTCGTGCTTTTCGCTGAAGAACTTGCGTTCTCGAGCGATACGAGCAAGACGGAAACCCGAGGTACGGGTATCCGCGAGCAGCCGCCCGTGCGGAGTCTTGGTGTATACCGAGACTGCCGTAGGCGGGCTGACTACGTAGTAGTCGGCAGCGAGGTCCGTCCCACCGAAGAACATCGAAGGCACCTGGGAGGAGAGCCGTACCCACACCTTGTAGAGGCGTGGATCTGACATACCTCCGACAACTCCCCAACCCCGTAGCCGATTTAATATCAGCATCAGGGCGAAGAGATTGTCAACAGGTTTCTTGATGTAAAACGGTTTGACATCGACACCACGGTAAAAGTGCGCGCCGCAGCTCTCGCGAAAGAGCCCGGACACGAACGTTTTACGAAGATTCGGTTTAAAACCGTAGTAGGCAAGTGCCTCTAGCACACGGGGTGCAATCTCACTGGGACATATAATATCGTCCCCGTAGATGCCTATGGTTCCGGCGTTACCAAAATGGATTTGGGTCGCTTTGACTATTGCCCAGAATATCATGGACTCTAGCTCAAATGTGAACCCATTTCCCATTGTGGAAAATAGTTCCCATCGTATCGTCTCGCCATCTACGATTCCGTAGTGTGAGCGGATACGATCGAGATATGAATATAGCTCAGGTGGGAGAAAACTCCACACCAGGCGATCGGAGATGGAATCGGATGCAGACGATAAGTCTATCGTCGCAAGCGAACCATCTACGCTGCCCTGCTGAGCCAGACGCTGGTTGATCGATTGATCATTTAGGTCTATACCAACGGATTTGAGCCGGCGTCTGATAAAGGCACCGACCCCTTTCTGGAGGTACATATTCATATCAGGCTCCTTACAGGCAGCCCGATCTATTTTATTATTCTTCGGAACTGTAAACACTCCGTTCCCTACAACGAGCCTAAATTCATATGACTCGTTATAGCGGACCGCGTGTCTGATCCACGGCGCACATTGGTCTCGGACCAATAGAGCCGCTCTCAGAGCGCGGGGGGTAACGGTTGCTTGTTCAGCGAACTTCTTGTAAGGCGCTGCATCCTGCAACTTGTGCCCCATAGAGGCACCGTTGGAGAACGTGCATTGCCCAAACAACGACGATCGGTAGCCAGAGAGGAGGTTGCCAATAAGGCTACGGATGCTGGTTTGTAAAACATCCGGATCCCATGACAAGGATTTGTCATGTAAGAAACCTTCTCTATTTATCTGACCGCACCGATCATTCGCCTCCCGTAGCTTAGCGATAGCTAAGGTACGACGGGTCGCCTCGTCATTACCgGAACCTGAGGTCGGATGCTTTGTGAGCAATTCGTCCCTTAAGTAAGCAATTGCTGTAAAGTCGTCACTGTGCGGATCACCGCTTCCAGTAGCGACAGAAGCAATTGATTGGTAAATTTCGAGAGAAAGATCGCGAGGAAGATCAATACATAAAGAGTTGAACTTCTTTGTTGTCTTCGACATGGGTAATCCTCATGTTTGAATGGCCGGCGTCTATTAGTAGATGCCGGAGTTTGCTGCGATTGCTGAGGGAATCGGGTTTCCATCTTTTAGGAGACCTTGCATTGCCTTAACAATAAGCTCGCAGTCGGAATTCGTAGCGAAAATTGGAATGGTTAGTTCCATATTTAAGTACGAACGCCATGCGGCTACAGGAAGCTCTACACCACCAACAGTCTGGGTTGCCACTTTAGGCACCTCGACTTTGATGGTGTATTTGCGATTCTGCGCAGAGCTCTGACGAACGCTACAGGTTACTTTGTAAGCCTGTGAACGCGAGTTAGAGCTGATCCATTCAGCGACCCCGTTAGCGAAGTTGCTTGGGGCGACAGTCACGTCGCCAGTTCCGCCATTGTCGACGAGAACGAACTGAGTAAAGTTAGAAGCCATGCTTCAAACTCCGGTTGAGGGCCCTCTAGATAG**AGACGGTCGGGTCCAGATATTCGTATCTGTCGAGTAGAGTGTGGGCT**CTATCTAGAGAGCCGTTGCCTGATTAATGCTAACGCATCTAAGGTATGGACCATCGAGAAAGGAGACTTTACGTACGCGCCAGTTGTTGGCCATACGGATTGTACCCCTCGATGCATGGCTGAGATTTGGGCCTTAGCAGTGCCCTGTCTCTCCACAGTCCACCCGTAGGGAGCGTCAACGCTTATGATGGACTCACCCGTTATTACGTCAGTAACTGTTCCTGACATGTAGGAGCATCCCACGGGGGCCGTAAGGCCCTCGAGCATGTTACCTACAGGTAGGAGCCAGTCGACAACGAATGAGAAAGGCACCTTTTCCCACACTATACCTAGTGGGTTCAAGATACCTAGAGACGACAACCATGCCAAACGTGCATCGTTTATGTAAAACCATATCACGATACGTCGCGATATGTTGCACGTTGTCTGGAAGTTTGCAGCTGGATACGACAGACGGCCATCTAACTTGATGTTAGTACCGACCTGACGTACGGCTCTCATAGGAAGAAACTCTTGAAGGTGAACCTTCGTAAGCATCTCATATGCACCCTGGATATCACTCATTAGTGGTAACCAACCGAACTGCAACTCCAACCACCTGCCGGCCACGTGTTTTGATCGAAACTTTCGATCTTCGTTTAGGGCAAGGTAGCGGAGCGCCTGGCGCCAATTACCGCGACGAGCGGCAGTGTACGCCTTCACGAGCGCAATGGTTTGCGTCGCGAGTTGTGAGGCTGTCGACCTGGCCTCTGCTAGAGCAACACCAAGGTTAAAACTACCCTGGGTGACCTTTTGCAGGACTTCGGTCGACGCCCGGTTCGCAACGTTCTGCGGCACTTCGATGTAAGTCAAGTTTTGGCTTACAGGGAAGAGGCTGTAGCAGGAGCGTGCGTCGAGGGAGAAGCCGAAACCGGCTTTCTCATCGTACGGGCGACCCCACGATGACCCACTTCGCTTGTAGGCACCTTGATCTATCGATGTGACACTTAACGCCCCCCGTGAATACGGAGAGGGGTAGTGCCACTGTTTCGTTTTGGCCCCAGTCGAGTTAAAACGACCGGGAGTCCAGTTCGAACGATATTTTAAAGAGAATGAGTTATCTTCAGTCTCACCGTCCGCGTAAACGCGAACGGAGGGGACGAAGGTCTCGTTCTCCCTATCAAGGGTACTAAAAGCTCGCACAGGTCAAACCTCCTAGGAATGGAATTCCGGCTACCTACAGCGATAGCCATGGTAGCGTCTCGCTAAAGACATTAAAAATGGCATTAGCTCGACAGGAAGTTGAGCAGGACCCCGAAAGGGGTCCCACCC |
| **[F30-Bro(+)]_MS2(+)_** |
| T7 promoter **Broccoli core (+)**  TAATACGACTCACTATAGGGTGGGACCCCTTTCGGGGTCCTGCTCAACTTCCTGTCGAGCTAATGCCATTTTTAATGTCTTTAGCGAGACGCTACCATGGCTATCGCTGTAGGTAGCCGGAATTCCATTCCTAGGAGGTTTGACCTGTGCGAGCTTTTAGTACCCTTGATAGGGAGAACGAGACCTTCGTCCCCTCCGTTCGCGTTTACGCGGACGGTGAGACTGAAGATAACTCATTCTCTTTAAAATATCGTTCGAACTGGACTCCCGGTCGTTTTAACTCGACTGGGGCCAAAACGAAACAGTGGCACTACCCCTCTCCGTATTCACGGGGGGCGTTAAGTGTCACATCGATAGATCAAGGTGCCTACAAGCGAAGTGGGTCATCGTGGGGTCGCCCGTACGAtGAGAAAGCCGGTTTCGGCTTCTCCCTCGACGCACGCTCCTGCTACAGCCTCTTCCCTGTAAGCCAAAACTTGACTTACATCGAAGTGCCGCAGAACGTTGCGAACCGGGCGTCGACCGAAGTCCTGCAAAAGGTCACCCAGGGTAGTTTTAACCTTGGTGTTGCTCTAGCAGAGGCCAGGTCGACAGCCTCACAACTCGCGACGCAAACCATTGCGCTCGTGAAGGCGTACACTGCCGCTCGTCGCGGTAATTGGCGCCAGGCGCTCCGCTACCTTGCCCTAAACGAAGATCGAAAGTTTCGATCAAAACACGTGGCCGGCAGGTGGTTGGAGTTGCAGTTCGGTTGGTTACCACTAATGAGTGATATCCAGGGTGCATATGAGATGCTTACGAAGGTTCACCTTCAAGAGTTTCTTCCTATGAGAGCCGTACGTCAGGTCGGTACTAACATCAAGTTAGATGGCCGTCTGTCGTATCCAGCTGCAAACTTCCAGACAACGTGCAACATATCGCGACGTATCGTGATATGGTTTTACATAAACGATGCACGTTTGGCATGGTTGTCGTCTCTAGGTATCTTGAACCCACTAGGTATAGTGTGGGAAAAGGTGCCTTTCTCATTCGTTGTCGACTGGCTCCTACCTGTAGGTAACATGCTCGAGGGCCTTACGGCCCCCGTGGGATGCTCCTACATGTCAGGAACAGTTACTGACGTAATAACGGGTGAGTCCATCATAAGCGTTGACGCTCCCTACGGGTGGACTGTGGAGAGACAGGGCACTGCTAAGGCCCAAATCTCAGCCATGCATCGAGGGGTACAATCCGTATGGCCAACAACTGGCGCGTACGTAAAGTCTCCTTTCTCGATGGTCCATACCTTAGATGCGTTAGCATTAATCAGGCAACGGCTCTCTAGATAG**AGACGGTCGGGTCCAGATATTCGTATCTGTCGAGTAGAGTGTGGGCT**CTATCTAGAGGGCCCTCAACCGGAGTTTGAAGCATGGCTTCTAACTTTACTCAGTTCGTTCTCGTCGACAATGGCGGAACTGGCGACGTGACTGTCGCCCCAAGCAACTTCGCTAACGGGGTCGCTGAATGGATCAGCTCTAACTCGCGTTCACAGGCTTACAAAGTAACCTGTAGCGTTCGTCAGAGCTCTGCGCAGAATCGCAAATACACCATCAAAGTCGAGGTGCCTAAAGTGGCAACCCAGACTGTTGGTGGTGTAGAGCTTCCTGTAGCCGCATGGCGTTCGTACTTAAATATGGAACTAACCATTCCAATTTTCGCTACGAATTCCGACTGCGAGCTTATTGTTAAGGCAATGCAAGGTCTCCTAAAAGATGGAAACCCGATTCCCTCAGCAATCGCAGCAAACTCCGGCATCTACTAATAGACGCCGGCCATTCAAACATGAGGATTACCCATGTCGAAGACAACAAAGAAGTTCAACTCTTTATGTATTGATCTTCCTCGCGATCTTTCTCTCGAAATTTACCAATCAATTGCTTCTGTCGCTACTGGAAGCGGTGATCCGCACAGTGACGACTTTACAGCAATTGCTTACTTAAGGGACGAATTGCTCACAAAGCATCCGACCTCAGGTTCCGGTAATGACGAGGCGACCCGTCGTACCTTAGCTATCGCTAAGCTACGGGAGGCGAATGATCGGTGCGGTCAGATAAATAGAGAAGGTTTCTTACATGACAAATCCTTGTCATGGGATCCGGATGTTTTACAAACCAGCATCCGTAGCCTTATTGGCAACCTCCTCTCTGGCTACCGATCGTCGTTGTTTGGGCAATGCACGTTCTCCAACGGTGCCtCTATGGGGCACAAGTTGCAGGATGCAGCGCCTTACAAGAAGTTCGCTGAACAAGCAACCGTTACCCCCCGCGCTCTGAGAGCGGCTCTATTGGTCCGAGACCAATGTGCGCCGTGGATCAGACACGCGGTCCGCTATAACGAGTCATATGAATTTAGGCTCGTTGTAGGGAACGGAGTGTTTACAGTTCCGAAGAATAATAAAATAGATCGGGCTGCCTGTAAGGAGCCTGATATGAATATGTACCTCCAGAAAGGGGTCGGTGCCTTTATCAGACGCCGGCTCAAATCCGTTGGTATAGACCTAAATGATCAATCGATCAACCAGCGTCTGGCTCAGCAGGGCAGCGTAGATGGTTCGCTTGCGACGATAGACTTATCGTCTGCATCCGATTCCATCTCCGATCGCCTGGTGTGGAGTTTTCTCCCACCTGAGCTATATTCATATCTCGATCGTATCCGCTCACACTACGGAATCGTAGATGGCGAGACGATACGATGGGAACTATTTTCCACAATGGGAAATGGGTTCACATTTGAGCTAGAGTCCATGATATTCTGGGCAATAGTCAAAGCGACCCAAATCCATTTTGGTAACGCCGGAACCATAGGCATCTACGGGGACGATATTATATGTCCCAGTGAGATTGCACCCCGTGTGCTAGAGGCACTTGCCTACTACGGTTTTAAACCGAATCTTCGTAAAACGTTCGTGTCCGGGCTCTTTCGCGAGAGCTGCGGCGCGCACTTTTACCGTGGTGTCGATGTCAAACCGTTTTACATCAAGAAACCTGTTGACAATCTCTTCGCCCTGATGCTGATATTAAATCGGCTACGGGGTTGGGGAGTTGTCGGAGGTATGTCAGATCCACGCCTCTACAAGGTGTGGGTACGGCTCTCCTCCCAGGTGCCTTCGATGTTCTTCGGTGGGACGGACCTCGCTGCCGACTACTACGTAGTCAGCCCGCCTACGGCAGTCTCGGTATACACCAAGACTCCGcACGGGCGGCTGCTCGCGGATACCCGTACCTCGGGTTTCCGTCTTGCTCGTATCGCTCGAGAACGCAAGTTCTTCAGCGAAAAGCACGACAGTGGTCGCTACATAGCGTGGTTCCATACTGGAGGTGAAATCACCGACAGCATGAAGTCCGCCGGCGTGCGCGTTATACGCACTTCGGAGTGGCTAACGCCGGTTCCCACATTCCCTCAGGAGTGTGGGCCAGCGAGCTCTCCTCGGTAGCTGACCGAGGGACCCCCGTAAACGGGGTGGGTGTGCTCGAAAGAGCACGGGTCCGCGAAAGCGGTGGCTCCACCGAAAGGTGGGCGGGCTTCGGCCCAGGGACCTCCCCCTAAAGAGAGGACCCGGGATTCTCCCGATTTGGTAACTAGCTGCTTGGCTAGTTACCACCC |
| **[F30-Bro(-)]_MS2(-)_** |
| T7 promoter **Broccoli core (-)**  TAATACGACTCACTATAGGGTGGTAACTAGCCAAGCAGCTAGTTACCAAATCGGGAGAATCCCGGGTCCTCTCTTTAGGGGGAGGTCCCTGGGCCGAAGCCCGCCCACCTTTCGGTGGAGCCACCGCTTTCGCGGACCCGTGCTCTTTCGAGCACACCCACCCCGTTTACGGGGGTCCCTCGGTCAGCTACCGAGGAGAGCTCGCTGGCCCACACTCCTGAGGGAATGTGGGAACCGGCGTTAGCCACTCCGAAGTGCGTATAACGCGCACGCCGGCGGACTTCATGCTGTCGGTGATTTCACCTCCAGTATGGAACCACGCTATGTAGCGACCACTGTCGTGCTTTTCGCTGAAGAACTTGCGTTCTCGAGCGATACGAGCAAGACGGAAACCCGAGGTACGGGTATCCGCGAGCAGCCGCCCGTGCGGAGTCTTGGTGTATACCGAGACTGCCGTAGGCGGGCTGACTACGTAGTAGTCGGCAGCGAGGTCCGTCCCACCGAAGAACATCGAAGGCACCTGGGAGGAGAGCCGTACCCACACCTTGTAGAGGCGTGGATCTGACATACCTCCGACAACTCCCCAACCCCGTAGCCGATTTAATATCAGCATCAGGGCGAAGAGATTGTCAACAGGTTTCTTGATGTAAAACGGTTTGACATCGACACCACGGTAAAAGTGCGCGCCGCAGCTCTCGCGAAAGAGCCCGGACACGAACGTTTTACGAAGATTCGGTTTAAAACCGTAGTAGGCAAGTGCCTCTAGCACACGGGGTGCAATCTCACTGGGACATATAATATCGTCCCCGTAGATGCCTATGGTTCCGGCGTTACCAAAATGGATTTGGGTCGCTTTGACTATTGCCCAGAATATCATGGACTCTAGCTCAAATGTGAACCCATTTCCCATTGTGGAAAATAGTTCCCATCGTATCGTCTCGCCATCTACGATTCCGTAGTGTGAGCGGATACGATCGAGATATGAATATAGCTCaGGTGGGAGAAAACTCCACACCAGGCGATCGGAGATGGAATCGGATGCAGACGATAAGTCTATCGTCGCAAGCGAACCATCTACGCTGCCCTGCTGAGCCAGACGCTGGTTGATCGATTGATCATTTAGGTCTATACCAACGGATTTGAGCCGGCGTCTGATAAAgGCACCGACCCCTTTCTGGAGGTACATATTCATATCAGGCTCCTTACAGGCAGCCCGATCTATTTTATTATTCTTCGGAACTGTAAACACTCCGTTCCCTACAACGAGCCTAAATTCATATGACTCGTTATAGCGGACCGCGTGTCTGATCCACGGCGCACATTGGTCTCGGACCAATAGAGCCGCTCTCAGAGCGCGGGGGGTAACGGTTGCTTGTTCAGCGAACTTCTTGTAAGGCGCTGCATCCTGCAACTTGTGCCCCATAGAGGCACCGTTGGAGAACGTGCATTGCCCAAACAACGACGATCGGTAGCCAGAGAGGAGGTTGCCAATAAGGCTACGGATGCTGGTTTGTAAAACATCCGGATCCCATGACAAGGATTTGTCATGTAAGAAACCTTCTCTATTTATCTGACCGCACCGATCATTCGCCTCCCGTAGCTTAGCGATAGCTAAGGTACGACGGGTCGCCTCGTCATTACCGGAACCTGAGGTCGGATGCTTTGTGAGCAATTCGTCCCTTAAGTAAGCAATTGCTGTAAAGTCGTCACTGTGCGGATCACCGCTTCCAGTAGCGACAGAAGCAATTGATTGGTAAATTTCGAGAGAAAGATCGCGAGGAAGATCAATACATAAAGAGTTGAACTTCTTTGTTGTCTTCGACATGGGTAATCCTCATGTTTGAATGGCCGGCGTCTATTAGTAGATGCCGGAGTTTGCTGCGATTGCTGAGGGAATCGGGTTTCCATCTTTTAGGAGACCTTGCATTGCCTTAACAATAAGCTCGCAGTCGGAATTCGTAGCGAAAATTGGAATGGTTAGTTCCATATTTAAGTACGAACGCCATGCGGCTACAGGAAGCTCTACACCACCAACAGTCTGGGTTGCCACTTTAGGCACCTCGACTTTGATGGTGTATTTGCGATTCTGCGCAGAGCTCTGACGAACGCTACAGGTTACTTTGTAAGCCTGTGAACGCGAGTTAGAGCTGATCCATTCAGCGACCCCGTTAGCGAAGTTGCTTGGGGCGACAGTCACGTCGCCAGTTCCGCCATTGTCGACGAGAACGAACTGAGTAAAGTTAGAAGCCATGCTTCAAACTCCGGTTGAGGGCCCTCTAGATAG**AGCCCACACTCTACTCGACAGATACGAATATCTGGACCCGACCGTCT**CTATCTAGAGAGCCGTTGCCTGATTAATGCTAACGCATCTAAGGTATGGACCATCGAGAAAGGAGACTTTACGTACGCGCCAGTTGTTGGCCATACGGATTGTACCCCTCGATGCATGGCTGAGATTTGGGCCTTAGCAGTGCCCTGTCTCTCCACAGTCCACCCGTAGGGAGCGTCAACGCTTATGATGGACTCACCCGTTATTACGTCAGTAACTGTTCCTGACATGTAGGAGCATCCCACGGGGGCCGTAAGGCCCTCGAGCATGTTACCTACAGGTAGGAGCCAGTCGACAACGAATGAGAAAGGCACCTTTTCCCACACTATACCTAGTGGGTTCAAGATACCTAGAGACGACAACCATGCCAAACGTGCATCGTTTATGTAAAACCATATCACGATACGTCGCGATATGTTGCACGTTGTCTGGAAGTTTGCAGCTGGATACGACAGACGGCCATCTAACTTGATGTTAGTACCGACCTGACGTACGGCTCTCATAGGAAGAAACTCTTGAAGGTGAACCTTCGTAAGCATCTCATATGCACCCTGGATATCACTCATTAGTGGTAACCAACCGAACTGCAACTCCAACCACCTGCCGGCCACGTGTTTTGATCGAAACTTTCGATCTTCGTTTAGGGCAAGGTAGCGGAGCGCCTGGCGCCAATTACCGCGACGAGCGGCAGTGTACGCCTTCACGAGCGCAATGGTTTGCGTCGCGAGTTGTGAGGCTGTCGACCTGGCCTCTGCTAGAGCAACACCAAGGTTAAAACTACCCTGGGTGACCTTTTGCAGGACTTCGGTCGACGCCCGGTTCGCAACGTTCTGCGGCACTTCGATGTAAGTCAAGTTTTGGCTTACAGGGAAGAGGCTGTAGCAGGAGCGTGCGTCGAGGGAGAAGCCGAAACCGGCTTTCTCATCGTACGGGCGACCCCACGATGACCCACTTCGCTTGTAGGCACCTTGATCTATCGATGTGACACTTAACGCCCCCCGTGAATACGGAGAGGGGTAGTGCCACTGTTTCGTTTTGGCCCCAGTCGAGTTAAAACGACCGGGAGTCCAGTTCGAACGATATTTTAAAGAGAATGAGTTATCTTCAGTCTCACCGTCCGCGTAAACGCGAACGGAGGGGACGAAGGTCTCGTTCTCCCTATCAAGGGTACTAAAAGCTCGCACAGGTCAAACCTCCTAGGAATGGAATTCCGGCTACCTACAGCGATAGCCATGGTAGCGTCTCGCTAAAGACATTAAAAATGGCATTAGCTCGACAGGAAGTTGAGCAGGACCCCGAAAGGGGTCCCACCC |
| **MS2wt(+)** |
| T7 promoter  TAATACGACTCACTATAGGGTGGGACCCCTTTCGGGGTCCTGCTCAACTTCCTGTCGAGCTAATGCCATTTTTAATGTCTTTAGCGAGACGCTACCATGGCTATCGCTGTAGGTAGCCGGAATTCCATTCCTAGGAGGTTTGACCTGTGCGAGCTTTTAGTACCCTTGATAGGGAGAACGAGACCTTCGTCCCCTCCGTTCGCGTTTACGCGGACGGTGAGACTGAAGATAACTCATTCTCTTTAAAATATCGTTCGAACTGGACTCCCGGTCGTTTTAACTCGACTGGGGCCAAAACGAAACAGTGGCACTACCCCTCTCCGTATTCACGGGGGGCGTTAAGTGTCACATCGATAGATCAAGGTGCCTACAAGCGAAGTGGGTCATCGTGGGGTCGCCCGTACGAtGAGAAAGCCGGTTTCGGCTTCTCCCTCGACGCACGCTCCTGCTACAGCCTCTTCCCTGTAAGCCAAAACTTGACTTACATCGAAGTGCCGCAGAACGTTGCGAACCGGGCGTCGACCGAAGTCCTGCAAAAGGTCACCCAGGGTAGTTTTAACCTTGGTGTTGCTCTAGCAGAGGCCAGGTCGACAGCCTCACAACTCGCGACGCAAACCATTGCGCTCGTGAAGGCGTACACTGCCGCTCGTCGCGGTAATTGGCGCCAGGCGCTCCGCTACCTTGCCCTAAACGAAGATCGAAAGTTTCGATCAAAACACGTGGCCGGCAGGTGGTTGGAGTTGCAGTTCGGTTGGTTACCACTAATGAGTGATATCCAGGGTGCATATGAGATGCTTACGAAGGTTCACCTTCAAGAGTTTCTTCCTATGAGAGCCGTACGTCAGGTCGGTACTAACATCAAGTTAGATGGCCGTCTGTCGTATCCAGCTGCAAACTTCCAGACAACGTGCAACATATCGCGACGTATCGTGATATGGTTTTACATAAACGATGCACGTTTGGCATGGTTGTCGTCTCTAGGTATCTTGAACCCACTAGGTATAGTGTGGGAAAAGGTGCCTTTCTCATTCGTTGTCGACTGGCTCCTACCTGTAGGTAACATGCTCGAGGGCCTTACGGCCCCCGTGGGATGCTCCTACATGTCAGGAACAGTTACTGACGTAATAACGGGTGAGTCCATCATAAGCGTTGACGCTCCCTACGGGTGGACTGTGGAGAGACAGGGCACTGCTAAGGCCCAAATCTCAGCCATGCATCGAGGGGTACAATCCGTATGGCCAACAACTGGCGCGTACGTAAAGTCTCCTTTCTCGATGGTCCATACCTTAGATGCGTTAGCATTAATCAGGCAACGGCTCTCTAGATAGgGCCCTCAACCGGAGTTTGAAGCATGGCTTCTAACTTTACTCAGTTCGTTCTCGTCGACAATGGCGGAACTGGCGACGTGACTGTCGCCCCAAGCAACTTCGCTAACGGGGTCGCTGAATGGATCAGCTCTAACTCGCGTTCACAGGCTTACAAAGTAACCTGTAGCGTTCGTCAGAGCTCTGCGCAGAATCGCAAATACACCATCAAAGTCGAGGTGCCTAAAGTGGCAACCCAGACTGTTGGTGGTGTAGAGCTTCCTGTAGCCGCATGGCGTTCGTACTTAAATATGGAACTAACCATTCCAATTTTCGCTACGAATTCCGACTGCGAGCTTATTGTTAAGGCAATGCAAGGTCTCCTAAAAGATGGAAACCCGATTCCCTCAGCAATCGCAGCAAACTCCGGCATCTACTAATAGACGCCGGCCATTCAAACATGAGGATTACCCATGTCGAAGACAACAAAGAAGTTCAACTCTTTATGTATTGATCTTCCTCGCGATCTTTCTCTCGAAATTTACCAATCAATTGCTTCTGTCGCTACTGGAAGCGGTGATCCGCACAGTGACGACTTTACAGCAATTGCTTACTTAAGGGACGAATTGCTCACAAAGCATCCGACCTCAGGTTCCGGTAATGACGAGGCGACCCGTCGTACCTTAGCTATCGCTAAGCTACGGGAGGCGAATGATCGGTGCGGTCAGATAAATAGAGAAGGTTTCTTACATGACAAATCCTTGTCATGGGATCCGGATGTTTTACAAACCAGCATCCGTAGCCTTATTGGCAACCTCCTCTCTGGCTACCGATCGTCGTTGTTTGGGCAATGCACGTTCTCCAACGGTGCCTCTATGGGGCACAAGTTGCAGGATGCAGCGCCTTACAAGAAGTTCGCTGAACAAGCAACCGTTACCCCCCGCGCTCTGAGAGCGGCTCTATTGGTCCGAGACCAATGTGCGCCGTGGATCAGACACGCGGTCCGCTATAACGAGTCATATGAATTTAGGCTCGTTGTAGGGAACGGAGTGTTTACAGTTCCGAAGAATAATAAAATAGATCGGGCTGCCTGTAAGGAGCCTGATATGAATATGTACCTCCAGAAAGGGGTCGGTGCCTTTATCAGACGCCGGCTCAAATCCGTTGGTATAGACCTAAATGATCAATCGATCAACCAGCGTCTGGCTCAGCAGGGCAGCGTAGATGGTTCGCTTGCGACGATAGACTTATCGTCTGCATCCGATTCCATCTCCGATCGCCTGGTGTGGAGTTTTCTCCCACCTGAGCTATATTCATATCTCGATCGTATCCGCTCACACTACGGAATCGTAGATGGCGAGACGATACGATGGGAACTATTTTCCACAATGGGAAATGGGTTCACATTTGAGCTAGAGTCCATGATATTCTGGGCAATAGTCAAAGCGACCCAAATCCATTTTGGTAACGCCGGAACCATAGGCATCTACGGGGACGATATTATATGTCCCAGTGAGATTGCACCCCGTGTGCTAGAGGCACTTGCCTACTACGGTTTTAAACCGAATCTTCGTAAAACGTTCGTGTCCGGGCTCTTTCGCGAGAGCTGCGGCGCGCACTTTTACCGTGGTGTCGATGTCAAACCGTTTTACATCAAGAAACCTGTTGACAATCTCTTCGCCCTGATGCTGATATTAAATCGGCTACGGGGTTGGGGAGTTGTCGGAGGTATGTCAGATCCACGCCTCTACAAGGTGTGGGTACGGCTCTCCTCCCAGGTGCCTTCGATGTTCTTCGGTGGGACGGACCTCGCTGCCGACTACTACGTAGTCAGCCCGCCTACGGCAGTCTCGGTATACACCAAGACTCCGCACGGGCGGCTGCTCGCGGATACCCGTACCTCGGGTTTCCGTCTTGCTCGTATCGCTCGAGAACGCAAGTTCTTCAGCGAAAAGCACGACAGTGGTCGCTACATAGCGTGGTTCCATACTGGAGGTGAAATCACCGACAGCATGAAGTCCGCCGGCGTGCGCGTTATACGCACTTCGGAGTGGCTAACGCCGGTTCCCACATTCCCTCAGGAGTGTGGGCCAGCGAGCTCTCCTCGGTAGCTGACCGAGGGACCCCCGTAAACGGGGTGGGTGTGCTCGAAAGAGCACGGGTCCGCGAAAGCGGTGGCTCCACCGAAAGGTGGGCGGGCTTCGGCCCAGGGACCTCCCCCTAAAGAGAGGACCCGGGATTCTCCCGATTTGGTAACTAGCTGCTTGGCTAGTTACCACCC |
| **MSRP-22 anti** |
| T7 promoter  TAATACGACTCACTATAAGGGGTGGTAACTAGCCAATCAGCTAGTTACCAAACCGGTAGAATCCCGGGTCCTCTCTTTAGGGGGAGGTCCCTGGGCCGAAGCCCCGCCCACCTTTCGGTGGAGCCCTAGGAATGGAATTCCGGCTACCTACAGCGATAGCCATGGTAGCGTCTCGCTAAAGACATTAAAAATGGCATTAGCTCGACAGGAAGTTGAGCAGGACCCCGAAAGGGGTCCCACCCA |
| **RQ-135-** |
| T7 promoter  TAATACGACTCACTATAGGGGTTCCAACCGGAAGTTGAGGGATGCCTAGGCATCCCCCGTGCGTCCCTTCGATCCTACGAGGGATTTGAGAGATGCCTAGGCATCTCCCGCGCGCCGGTTTCGGACCTCCAGTGCGTGTTACCGCACTGTTAGCCC |
