# Supplementary material for: In vitro characterisation of the MS2 RNA polymerase complex reveals host factors that modulate emesviral replicase activity
Source: Commun Biol. 2022 Mar 25;5:264. doi: 10.1038/s42003-022-03178-2 (PMC8956599; doi:10.1038/s42003-022-03178-2)
Supplement: Supplementary file 8 — Supplementary Data 5 [file 42003_2022_3178_MOESM8_ESM.docx]

| **pBAD33 backbone** |
| --- |
| *CmR* P15A ori **araBp**  TTTTGTATAGAATTTACGAAGCTTGGCTGTTTTGGCGGATGAGAGAAGATTTTCAGCCTGATACAGATTAAATCAGAACGCAGAAGCGGTCTGATAAAACAGAATTTGCCTGGCGGCAGTAGCGCGGTGGTCCCACCTGACCCCATGCCGAACTCAGAAGTGAAACGCCGTAGCGCCGATGGTAGTGTGGGGTCTCCCCATGCGAGAGTAGGGAACTGCCAGGCATCAAATAAAACGAAAGGCTCAGTCGAAAGACTGGGCCTTTCGTTTTATCTGTTGTTTGTCGGTGAACGCTCTCCTGAGTAGGACAAATCCGCCGGGAGCGGATTTGAACGTTGCGAAGCAACGGCCCGGAGGGTGGCGGGCAGGACGCCCGCCATAAACTGCCAGGCATCAAATTAAGCAGAAGGCCATCCTGACGGATGGCCTTTTTGCGTTTCTACAAACTCTTTTGTTTATTTTTCTAAATACATTCAAATATGTATCCGCTCATGAGACAATAACCCTGATAAATGCTTCAATAATATTGAAAAAGGAAGAGTATGAGTATTCAACATTTCCGTGTCGCCCTTATTCCCTTTTTTGCGGCATTTTGCCTTCCTGTTTTTGCTCACCCAGAAACGCTGGTGAAAGTAAAAGATGCTGAAGATCAGTTGGGTGCAGCAAACTATTAACTGGCGAACTACTTACTCTAGCTTCCCGGCAACAATTAATAGACTGGATGGAGGCGGATAAAGTTGCAGGACCACTTCTGCGCTCGGCCCTTCCGGCTGGCTGGTTTATTGCTGATAAATCTGGAGCCGGTGAGCGTGGGTCTCGCGGTATCATTGCAGCACTGGGGCCAGATGGTAAGCCCTCCCGTATCGTAGTTATCTACACGACGGGGAGTCAGGCAACTATGGATGAACGAAATAGACAGATCGCTGAGATAGGTGCCTCACTGATTAAGCATTGGTAACTGTCAGACCAAGTTTACTCATATATACTTTAGATTGATTTACGCGCCCTGTAGCGGCGCATTAAGCGCGGCGGGTGTGGTGGTTACGCGCAGCGTGACCGCTACACTTGCCAGCGCCCTAGCGCCCGCTCCTTTCGCTTTCTTCCCTTCCTTTCTCGCCACGTTCGCCGGCTTTCCCCGTCAAGCTCTAAATCGGGGGCTCCCTTTAGGGTTCCGATTTAGTGCTTTACGGCACCTCGACCCCAAAAAACTTGATTTGGGTGATGGTTCACGTAGTGGGCCATCGCCCTGATAGACGGTTTTTCGCCCTTTGACGTTGGAGTCCACGTTCTTTAATAGTGGACTCTTGTTCCAAACTTGAACAACACTCAACCCTATCTCGGGCTATTCTTTTGATTTATAAGGGATTTTGCCGATTTCGGCCTATTGGTTAAAAAATGAGCTGATTTAACAAAAATTTAACGCGAATTTTAACAAAATATTAACGTTTACAATTTAAAAGGATCTAGGTGAAGATCCTTTTTGATAATCTCATGACCAAAATCCCTTAACGTGAGTTTTCGTTCCACTGAGCGTCAGACCCCGTAGAAAAGATCAAAGGATCTTCTTGAGATCCTTTTTTTCTGCGCGTAATCTGCTGCTTGCAAACAAAAAAACCACCGCTACCAGCGGTGGTTTGTTTGCCGGATCAAGAGCTACCAACTCTTTTTCCGAAGGTAACTGGCTTCAGCAGAGCGCAGATACCAAATACTGTCCTTCTAGTGTAGCCGTAGTTAGGCCACCACTTCAAGAACTCTGTAGCACCGCCTACATACCTCGCTCTGCTAATCCTGTTACCAGTCAGGCATTTGAGAAGCACACGGTCACACTGCTTCCGGTAGTCAATAAACCGGTAAACCAGCAATAGACATAAGCGGCTATTTAACGACCCTGCCCTGAACCGACGACCGGGTCGAATTTGCTTTCGAATTTCTGCCATTCATCCGCTTATTATCACTTATTCAGGCGTAGCACCAGGCGTTTAAGGGCACCAATAACTGCCTTAAAAAAATTA*CGCCCCGCCCTGCCACTCATCGCAGTACTGTTGTAATTCATTAAGCATTCTGCCGACATGGAAGCCATCACAGACGGCATGATGAACCTGAATCGCCAGCGGCATCAGCACCTTGTCGCCTTGCGTATAATATTTGCCCATGGTGAAAACGGGGGCGAAGAAGTTGTCCATATTGGCCACGTTTAAATCAAAACTGGTGAAACTCACCCAGGGATTGGCTGAGACGAAAAACATATTCTCAATAAACCCTTTAGG*GAAATAGGCCAGGTTTTCACCGTAACACGCCACATCTTGCGAATATATGTGTAGAAACTGCCGGAAATCGTCGTGGTATTCACTCCAGAGCGATGAAAACGTTTCAGTTTGCTCATGGAAAACGGTGTAACAAGGGTGAACACTATCCCATATCACCAGCTCACCGTCTTTCATTGCCATACGGAATTCCGGATGAGCATTCATCAGGCGGGCAAGAATGTGAATAAAGGCCGGATAAAACTTGTGCTTATTTTTCTTTACGGTCTTTAAAAAGGCCGTAATATCCAGCTGAACGGTCTGGTTATAGGTACATTGAGCAACTGACTGAAATGCCTCAAAATGTTCTTTACGATGCCATTGGGATATATCAACGGTGGTATATCCAGTGATTTTTTTCTCCATTTTAGCTTCCTTAGCTCCTGAAAATCTCGATAACTCAAAAAATACGCCCGGTAGTGATCTTATTTCATTATGGTGAAAGTTGGAACCTCTTACGTGCCGATCAACGTCTCATTTTCGCCAAAAGTTGGCCCAGGGCTTCCCGGTATCAACAGGGACACCAGGATTTATTTATTCTGCGAAGTGATCTTCCGTCACAGGTATTTATTCGGCGCAAAGTGCGTCGGGTGATGCTGCCAACTTACTGATTTAGTGTATGATGGTGTTTTTGAGGTGCTCCAGTGGCTTCTGTTTCTATCAGCTGTCCCTCCTGTTCAGCTACTGACGGGGTGGTGCGTAACGGCAAAAGCACCGCCGGACATCAGCGCTAGCGGAGTGTATACTGGCTTACTATGTTGGCACTGATGAGGGTGTCAGTGAAGTGCTTCATGTGGCAGGAGAAAAAAGGCTGCACCGGTGCGTCAGCAGAATATGTGATACAGGATATATTCCGCTTCCTCGCTCACTGACTCGCTACGCTCGGTCGTTCGACTGCGGCGAGCGGAAATGGCTTACGAACGGGGCGGAGATTTCCTGGAAGATGCCAGGAAGATACTTAACAGGGAAGTGAGAGGGCCGCGGCAAAGCCGTTTTTCCATAGGCTCCGCCCCCCTGACAAGCATCACGAAATCTGACGCTCAAATCAGTGGTGGCGAAACCCGACAGGACTATAAAGATACCAGGCGTTTCCCCCTGGCGGCTCCCTCGTGCGCTCTCCTGTTCCTGCCTTTCGGTTTACCGGTGTCATTCCGCTGTTATGGCCGCGTTTGTCTCATTCCACGCCTGACACTCAGTTCCGGGTAGGCAGTTCGCTCCAAGCTGGACTGTATGCACGAACCCCCCGTTCAGTCCGACCGCTGCGCCTTATCCGGTAACTATCGTCTTGAGTCCAACCCGGAAAGACATGCAAAAGCACCACTGGCAGCAGCCACTGGTAATTGATTTAGAGGAGTTAGTCTTGAAGTCATGCGCCGGTTAAGGCTAAACTGAAAGGACAAGTTTTGGTGACTGCGCTCCTCCAAGCCAGTTACCTCGGTTCAAAGAGTTGGTAGCTCAGAGAACCTTCGAAAAACCGCCCTGCAAGGCGGTTTTTTCGTTTTCAGAGCAAGAGATTACGCGCAGACCAAAACGATCTCAAGAAGATCATCTTATTAATCAGATAAAATATTTCTAGGCTCATGAGCCCGAAGTGGCGAGCCCGATCTTCCCCATCGGTGATGTCGGCGATATAGGCGCCAGCAACCGCACCTGTGGCGCCGGTGATGCCGGCCACGATGCGTCCGGCGTAGAGGATCTGCTCATGTTTGACAGCTTATCATCGATGCATAATGTGCCTGTCAAATGGACGAAGCAGGGATTCTGCAAACCCTATGCTACTCCGTCAAGCCGTCAATTGTCTGATTCGTTACCAATTATGACAACTTGACGGCTACATCATTCACTTTTTCTTCACAACCGGCACGGAACTCGCTCGGGCTGGCCCCGGTGCATTTTTTAAATACCCGCGAGAAATAGAGTTGATCGTCAAAACCAACATTGCGACCGACGGTGGCGATAGGCATCCGGGTGGTGCTCAAAAGCAGCTTCGCCTGGCTGATACGTTGGTCCTCGCGCCAGCTTAAGACGCTAATCCCTAACTGCTGGCGGAAAAGATGTGACAGACGCGACGGCGACAAGCAAACATGCTGTGCGACGCTGGCGATATCAAAATTGCTGTCTGCCAGGTGATCGCTGATGTACTGACAAGCCTCGCGTAC CCGATTATCCATCGGTGGATGGAGCGACTCGTTAATCGCTTCCATGCGCCGCAGTAACAATTGCTCAAGCAGATTTATCGCCAGCAGCTCCGAATAGCGCCCTTCCCCTTGCCCGGCGTTAATGATTTGCCCAAACAGGTCGCTGAAATGCGGCTGGTGCGCTTCATCCGGGCGAAAGAACCCCGTATTGGCAAATATTGACGGCCAGTTAAGCCATTCATGCCAGTAGGCGCGCGGACGAAAGTAAACCCACTGGTGATACCATTCGCGAGCCTCCGGATGACGACCGTAGTGATGAATCTCTCCTGGCGGGAACAGCAAAATATCACCCGGTCGGCAAACAAATTCTCGTCCCTGATTTTTCACCACCCCCTGACCGCGAATGGTGAGATTGAGAATATAACCTTTCATTCCCAGCGGTCGGTCGATAAAAAAATCGAGATAACCGTTGGCCTCAATCGGCGTTAAACCCGCCACCAGATGGGCATTAAACGAGTATCCCGGCAGCAGGGGATCATTTTGCGCTTCAGCCATACTTTTCATACTCCCGCCATTCAGAGAAGAAACCAATTGTCCATATTGCATCAGACATTGCCGTCACTGCGTCTTTTACTGGCTCTTCTCGCTAACCAAACCGGTAACCCCGCTTATTAAAAGCATTCTGTAACAAAGCGGGACCAAAGCCATGACAAAAACGCGTAACAAAAGTGTCTATAATCACGGCAGAAAAGTCCACATTGATTATTTGCACGGCGTCACACTTTGCTATGCCATAGCATTTTTATCCATAAGATTAGCGGATCCTACCTGACGCTTTTTATCGCAACTCTCTACT**GTTTCTCCATACCCGTTTTTTTGGGCTAGC**AGGAGGAATTCACC |
| **His_6_-MS2rep insert** |
| **Start** **Stop**  **ATG**CATCACCATCACCATCACTCGAAGACAACAAAGAAGTTCAACTCTTTATGTATTGATCTTCCTCGCGATCTTTCTCTCGAAATTTACCAATCAATTGCTTCTGTCGCTACTGGAAGCGGTGATCCGCACAGTGACGACTTTACAGCAATTGCTTACTTAAGGGACGAATTGCTCACAAAGCATCCGACCTTAGGTTCTGGTAATGACGAGGCGACCCGTCGTACCTTAGCTATCGCTAAGCTACGGGAGGCGAATGATCGGTGCGGTCAGATAAATAGAGAAGGTTTCTTACATGACAAATCCTTGTCATGGGATCCGGATGTTTTACAAACCAGCATCCGTAGCCTTATTGGCAACCTCCTCTCTGGCTACCGATCGTCGTTGTTTGGGCAATGCACGTTCTCCAACGGTGCCTCTATGGGGCACAAGTTGCAGGATGCAGCGCCCTACAAGAAGTTCGCTGAACAAGCAACCGTTACCCCCCGCGCTCTGAGAGCGGCTCTATTGGTCCGAGACCAATGTGCGCCGTGGATCAGACACGCGGTCCGCTATAACGAGTCATATGAGTTTAGGCTCGTTGTAGGGAACGGAGTGTTTACAGTTCCGAAGAATAATAAAATAGATCGGGCTGCCTGTAAGGAGCCTGATATGAATATGTACCTCCAGAAAGGGGTCGGTGCCTTTATCAGACGCCGGCTCAAATCCGTTGGTATAGACCTGAATGATCAATCGATCAACCAGCGTCTGGCTCAGCAGGGCAGCGTAGATGGTTCGCTTGCGACGATAGACTTATCGTCTGCATCCGATTCCATCTCCGATCGCCTGGTGTGGAGTTTTCTCCCACCTGAGCTATATTCATATCTCGATCGTATCCGCTCACACTACGGAATCGTAGATGGCGAGACGATACGATGGGAACTATTTTCCACAATGGGAAATGGGTTCACATTTGAGCTAGAGTCCATGATATTCTGGGCAATAGTCAAAGCGACCCAAATCCATTTTGGTAACGCCGGAACCATAGGCATCTACGGGGACGATATTATATGCCCCAGTGAGATTGCACCCCGTGTGCTAGAGGCACTTGCCTACTACGGTTTTAAACCGAATCTTCGCAAAACGTTCGTGTCCGGGCTCTTTCGCGAGAGCTGCGGCGCGCACTTTTACCGTGGTGTCGATGTCAAACCGTTTTACATCAAGAAACCTGTTGACAATCTCTTCGCCCTGATGCTGATATTAAATCGGCTACGGGGTTGGGGAGTTGTCGGAGGTATGTCAGATCCACGCCTCTACAAGGTGTGGGTACGGCTCTCCTCCCAGGTGCCTTCGATGTTCTTCGGTGGGACGGACCTCGCTGCCGACTACTACGTAGTCAGCCCGCCTACGGCAGTCTCGGTATACACCAAGACTCCGTACGGGCGGCTGCTCGCGGATACCCGTACCTCGGGTTTCCGTCTTGCTCGTATCGCTCGAGAACGCAAGTTCTTCAGCGAAAAGCACGACAGTGGTCGCTACATAGCGTGGTTCCATACTGGAGGTGAAATCACCGACAGCATGAAGTCCGCCGGCGTGCGCGTTATACGCACTTCGGAGTGGCTAACGCCGGTTCCCACATTCCCTCAGGAGTGTGGGCCAGCGAGCTCTCCTCGG**TAA** |
| **Qβrep-His_6_ insert** |
| **Start** **Stop**  **ATG**TCTAAGACAGCATCTTCGCGTAACTCTCTCAGCGCACAATTGCGCCGAGCCGCGAACACAAGAATTGAGGTTGAAGGTAACCTCGCACTTTCCATTGCCAACGATTTACTGTTGGCCTATGGTCAGTCGCCATTTAACTCTGAGGCTGAGTGTATTTCATTCAGCCCGAGATTCGACGGGACCCCGGATGACTTTAGGATAAATTATCTTAAAGCCGAGATCATGTCGAAGTATGACGACTTCAGCCTAGGTATTGATACCGAAGCTGTTGCCTGGGAGAAGTTCCTGGCAGCAGAGGCTGAATGTGCTTTAACGAACGCTCGTCTCTATAGGCCTGACTACAGTGAGGATTTCAATTTCTCACTGGGCGAGTCATGTATACACATGGCTCGTAGAAAAATAGCCAAGCTAATAGGAGATGTTCCGTCCGTTGAGGGTATGTTGCGTCACTGCCGATTTTCTGGCGGTGCTACAACAACGAATAACCGTTCGTACGGTCATCCGTCCTTCAAGTTTGCGCTTCCGCAAGCGTGTACGCCTCGGGCTTTGAAGTATGTTTTAGCTCTCAGAGCTTCTACACATTTCGATATCAGAATTTCTGATATTAGCCCTTTTAATAAAGCAGTTACTGTACCTAAGAACAGTAAGACAGATCGTTGTATTGCTATCGAACCTGGTTGGAATATGTTTTTCCAACTGGGTATCGGTGGCATTCTACGCGATCGGTTGCGTTGCTGGGGTATCGATCTGAATGATCAGACGATAAATCAGCGCCGCGCTCACGAAGGCTCCGTTACTAATAACTTAGCAACGGTTGATCTCTCAGCGGCAAGCGATTCTATATCTCTTGCCCTCTGTGAGCTCTTATTGCCCCCAGGCTGGTTTGAGGTTCTTATGGACCTCAGATCACCTAAGGGGCGATTGCCTGACGGTAGTGTTGTTACCTACGAGAAGATTTCTTCTATGGGTAACGGTTACACATTCGAGCTCGAGTCGCTTATTTTTGCTTCTCTCGCTCGTTCCGTTTGTGAGATACTGGACTTAGACTCGTCTGAGGTCACTGTTTACGGAGACGATATTATTTTACCGTCCTGTGCAGTCCCTGCCCTCCGGGAAGTTTTTAAGTATGTTGGTTTTACGACCAATACTAAAAAGACTTTTTCCGAGGGGCCGTTCAGAGAGTCGTGCGGCAAGCACTACTATTCTGGCGTAGATGTTACTCCCTTTTACATACGTCACCGTATAGTGAGTCCTGCCGATTTAATACTGGTTTTGAATAACCTATATCGGTGGGCCACAATTGACGGCGTATGGGATCCTAGGGCCCATTCTGTGTACCTCAAGTATCGTAAGTTGCTGCCTAAACAGCTGCAACGTAATACTATACCTGATGGTTACGGTGATGGTGCCCTCGTCGGATCGGTCCTAATCAATCCTTTCGCGAAAAACCGCGGGTGGATCCGGTACGTACCGGTGATTACGGACCATACAAGGGACCGAGAGCGCGCTGAGTTGGGGTCGTATCTCTACGACCTCTTCTCGCGTTGTCTCTCGGAAAGTAACGATGGGTTGCCTCTTAGGGGTCCATCGGGTTGCGATTCTGCGGATCTATTTGCCATCGATCAGCTTATCTGTAGGAGTAATCCTACGAAGATAAGCAGGTCTACCGGCAAATTCGATATACAGTATATCGCGTGCAGTAGCCGTGTTCTGGCACCCTACGGGGTCTTCCAGGGCACGAAGGTTGCGTCTCTACACGAGGCGCACCACCACCACCACCAC**TAA** |
| **EF-Tu-His_6_ insert** |
| **Start** **Stop**  **ATG**TCTAAAGAAAAGTTTGAACGTACAAAACCGCACGTTAACGTCGGTACTATCGGCCACGTTGACCATGGTAAAACAACGCTGACCGCTGCAATCACTACCGTACTGGCTAAAACCTACGGCGGTGCTGCTCGCGCATTCGACCAGATCGATAACGCGCCGGAAGAAAAAGCTCGTGGTATCACCATCAACACTTCTCACGTTGAATACGACACCCCGACCCGTCACTACGCACACGTAGACTGCCCGGGGCACGCCGACTATGTTAAAAACATGATCACCGGTGCTGCGCAGATGGACGGCGCGATCCTGGTAGTTGCTGCGACTGACGGCCCGATGCCGCAGACTCGTGAGCACATCCTGCTGGGTCGTCAGGTAGGCGTTCCGTACATCATCGTGTTCCTGAACAAATGCGACATGGTTGATGACGAAGAGCTGCTGGAACTGGTTGAAATGGAAGTTCGTGAACTTCTGTCTCAGTACGACTTCCCGGGCGACGACACTCCGATCGTTCGTGGTTCTGCTCTGAAAGCGCTGGAAGGCGACGCAGAGTGGGAAGCGAAAATCCTGGAACTGGCTGGCTTCCTGGATTCTTACATTCCGGAACCAGAGCGTGCGATTGACAAGCCGTTCCTGCTGCCGATCGAAGACGTATTCTCCATCTCCGGTCGTGGTACCGTTGTTACCGGTCGTGTAGAACGCGGTATCATCAAAGTTGGTGAAGAAGTTGAAATCGTTGGTATCAAAGAGACTCAGAAGTCTACCTGTACTGGCGTTGAAATGTTCCGCAAACTGCTGGACGAAGGCCGTGCTGGTGAGAACGTAGGTGTTCTGCTGCGTGGTATCAAACGTGAAGAAATCGAACGTGGTCAGGTACTGGCTAAGCCGGGCACCATCAAGCCGCACACCAAGTTCGAATCTGAAGTGTACATTCTGTCCAAAGATGAAGGCGGCCGTCATACTCCGTTCTTCAAAGGCTACCGTCCGCAGTTCTACTTCCGTACTACTGACGTGACTGGTACCATCGAACTGCCGGAAGGCGTAGAGATGGTAATGCCGGGCGACAACATCAAAATGGTTGTTACCCTGATCCACCCGATCGCGATGGACGACGGTCTGCGTTTCGCAATCCGTGAAGGCGGCCGTACCGTTGGCGCGGGCGTTGTAGCAAAAGTTCTGCACCACCACCACCACCAC**TAA** |
| **EF-Ts-His_6_ insert** |
| **Start** **Stop**  **ATG**GCTGAAATTACCGCATCCCTGGTAAAAGAGCTGCGTGAGCGTACTGGCGCAGGCATGATGGATTGCAAAAAAGCACTGACTGAAGCTAACGGCGACATCGAGCTGGCAATCGAAAACATGCGTAAGTCCGGTGCTATTAAAGCAGCGAAAAAAGCAGGCAACGTTGCTGCTGACGGCGTGATCAAAACCAAAATCGACGGCAACTACGGCATCATTCTGGAAGTTAACTGCCAGACTGACTTCGTTGCAAAAGACGCTGGTTTCCAGGCGTTCGCAGACAAAGTTCTGGACGCAGCTGTTGCTGGCAAAATCACTGACGTTGAAGTTCTGAAAGCACAGTTCGAAGAAGAACGTGTTGCGCTGGTAGCGAAAATTGGTGAAAACATCAACATTCGCCGCGTTGCTGCGCTGGAAGGCGACGTTCTGGGTTCTTATCAGCACGGTGCGCGTATCGGCGTTCTGGTTGCTGCTAAAGGCGCTGACGAAGAGCTGGTTAAACACATCGCTATGCACGTTGCTGCAAGCAAGCCAGAATTCATCAAACCGGAAGACGTATCCGCTGAAGTGGTAGAAAAAGAATACCAGGTACAGCTGGATATCGCGATGCAGTCTGGTAAGCCGAAAGAAATCGCGGAGAAAATGGTTGAAGGCCGCATGAAGAAATTCACCGGCGAAGTTTCTCTGACCGGTCAGCCGTTCGTTATGGAACCAAGCAAAACTGTTGGTCAGCTGCTGAAAGAGCATAACGCTGAAGTGACTGGCTTCATCCGCTTCGAAGTGGGTGAAGGCATCGAGAAAGTTGAGACTGACTTTGCAGCAGAAGTTGCTGCGATGTCCAAGCAGTCTCACCACCACCACCACCAC**TAA** |
| **S1-His_6_ insert** |
| **Start** **Stop**  **ATG**ACTGAATCTTTTGCTCAACTCTTTGAAGAGTCCTTAAAAGAAATCGAAACCCGCCCGGGTTCTATCGTTCGTGGCGTTGTTGTTGCTATCGACAAAGACGTAGTACTGGTTGACGCTGGTCTGAAATCTGAGTCCGCCATCCCGGCTGAGCAGTTCAAAAACGCCCAGGGCGAGCTGGAAATCCAGGTAGGTGACGAAGTTGACGTTGCTCTGGACGCAGTAGAAGACGGCTTCGGTGAAACTCTGCTGTCCCGTGAGAAAGCTAAACGTCACGAAGCCTGGATCACGCTGGAAAAAGCTTACGAAGATGCTGAAACTGTTACCGGTGTTATCAACGGCAAAGTTAAGGGCGGCTTCACTGTTGAGCTGAACGGTATTCGTGCGTTCCTGCCAGGTTCTCTGGTAGACGTTCGTCCGGTGCGTGACACTCTGCACCTGGAAGGCAAAGAGCTTGAATTTAAAGTAATCAAGCTGGATCAGAAGCGCAACAACGTTGTTGTTTCTCGTCGTGCCGTTATCGAATCCGAAAACAGCGCAGAGCGCGATCAGCTGCTGGAAAACCTGCAGGAAGGCATGGAAGTTAAAGGTATCGTTAAGAACCTCACTGACTACGGTGCATTCGTTGATCTGGGCGGCGTTGACGGCCTGCTGCACATCACTGACATGGCCTGGAAACGCGTTAAGCATCCGAGCGAAATCGTCAACGTGGGCGACGAAATCACTGTTAAAGTGCTGAAGTTCGACCGCGAACGTACCCGTGTATCCCTGGGCCTGAAACAGCTGGGCGAAGATCCGTGGGTAGCTATCGCTAAACGTTATCCGGAAGGTACCAAACTGACTGGTCGCGTGACCAACCTGACCGACTACGGCTGCTTCGTTGAAATCGAAGAAGGCGTTGAAGGCCTGGTACACGTTTCCGAAATGGACTGGACCAACAAAAACATCCACCCGTCCAAAGTTGTTAACGTTGGCGATGTAGTGGAAGTTATGGTTCTGGATATCGACGAAGAACGTCGTCGTATCTCCCTGGGTCTGAAACAGTGCAAAGCTAACCCGTGGCAGCAGTTCGCGGAAACCCACAACAAGGGCGACCGTGTTGAAGGTAAAATCAAGTCTATCACTGACTTCGGTATCTTCATCGGCTTGGACGGCGGCATCGACGGCCTGGTTCACCTGTCTGACATCTCCTGGAACGTTGCAGGCGAAGAAGCAGTTCGTGAATACAAAAAAGGCGACGAAATCGCTGCAGTTGTTCTGCAGGTTGACGCAGAACGTGAACGTATCTCCCTGGGCGTTAAACAGCTCGCAGAAGATCCGTTCAACAACTGGGTTGCTCTGAACAAGAAAGGCGCTATCGTAACCGGTAAAGTAACTGCAGTTGACGCTAAAGGCGCAACCGTAGAACTGGCTGACGGCGTTGAAGGTTACCTGCGTGCTTCTGAAGCATCCCGTGACCGCGTTGAAGACGCTACCCTGGTTCTGAGCGTTGGCGACGAAGTTGAAGCTAAATTCACCGGCGTTGATCGTAAAAACCGCGCAATCAGCCTGTCTGTTCGTGCGAAAGACGAAGCTGACGAGAAAGATGCAATCGCAACTGTTAACAAACAGGAAGATGCAAACTTCTCCAACAACGCAATGGCTGAAGCTTTCAAAGCAGCTAAAGGCGAGCACCACCACCACCACCACCAC**TAA** |
| **MTF-His_6_ insert** |
| **Start** **Stop**  **ATG**TCAGAATCACTACGTATTATTTTTGCGGGTACACCTGACTTTGCAGCGCGTCATCTCGACGCGCTGTTGTCTTCTGGTCATAACGTCGTTGGCGTGTTCACCCAGCCAGACCGACCGGCAGGACGCGGTAAAAAACTGATGCCCAGCCCGGTTAAAGTTCTGGCTGAGGAAAAAGGTCTGCCCGTTTTTCAACCTGTTTCCCTGCGTCCACAAGAAAACCAGCAACTGGTCGCCGAACTGCAAGCTGATGTTATGGTCGTCGTCGCCTATGGTTTAATTCTGCCGAAAGCAGTGCTGGAGATGCCGCGTCTTGGCTGTATCAACGTTCATGGTTCACTGCTGCCACGCTGGCGCGGTGCTGCACCAATCCAACGCTCACTATGGGCGGGTGATGCAGAAACTGGTGTGACCATTATGCAAATGGATGTCGGTTTAGACACCGGTGATATGCTCTATAAGCTCTCCTGCCCGATTACCGCAGAAGATACCAGTGGTACGCTGTACGACAAGCTGGCAGAGCTTGGCCCACAAGGGCTTATCACCACGTTGAAACAACTGGCAGACGGCACGGCGAAACCAGAAGTTCAGGACGAAACTCTTGTCACTTACGCCGAGAAGTTGAGTAAAGAAGAAGCGCGTATTGACTGGTCACTTTCGGCAGCACAGCTTGAACGCTGCATTCGCGCTTTCAATCCATGGCCAATGAGCTGGCTGGAAATTGAAGGACAGCCGGTTAAAGTCTGGAAAGCATCGGTCATTGATACGGCAACCAACGCTGCACCAGGAACGATCCTTGAAGCCAACAAACAAGGCATTCAGGTTGCGACTGGTGATGGCATCCTGAACCTGCTCTCGTTACAACCTGCGGGTAAGAAAGCGATGAGCGCGCAAGACCTCCTGAACTCTCGTCGGGAATGGTTTGTTCCGGGCAACCGTCTGGTCCACCACCACCACCACCAC**TAA** |
| **Ile-His_6_ insert** |
| **Start** **Stop**  **ATG**AGTGACTATAAATCAACCCTGAATCTGCCGGAAACAGGGTTCCCGATGCGTGGCGATCTCGCCAAGCGCGAACCCGGAATGCTGGCGCGTTGGACTGATGATGATCTGTACGGCATCATCCGTGCGGCTAAAAAAGGCAAAAAAACCTTCATTCTGCATGATGGCCCTCCTTATGCGAATGGCAGCATTCATATTGGTCACTCGGTTAACAAGATTCTGAAAGACATTATCGTGAAGTCCAAAGGGCTTTCCGGTTATGACTCGCCGTATGTGCCTGGCTGGGACTGCCACGGTCTGCCGATCGAGCTGAAAGTCGAGCAAGAATACGGTAAGCCGGGTGAGAAGTTCACCGCCGCCGAGTTCCGCGCCAAGTGCCGCGAATACGCGGCGACCCAGGTTGACGGTCAACGCAAAGACTTTATCCGTCTGGGCGTGCTGGGCGACTGGTCGCACCCGTACCTGACCATGGACTTCAAAACTGAAGCCAACATCATCCGCGCGCTGGGCAAAATCATCGGCAACGGTCACCTGCACAAAGGCGCGAAGCCAGTTCACTGGTGCGTTGACTGCCGTTCTGCGCTGGCGGAAGCGGAAGTTGAGTATTACGACAAAACTTCTCCGTCCATCGACGTTGCTTTCCAGGCAGTCGATCAGGATGCACTGAAAGCAAAGTTTGCCGTAAGCAACGTTAACGGCCCAATCTCGCTGGTAATCTGGACCACCACGCCGTGGACTCTGCCTGCCAACCGCGCAATCTCTATTGCACCAGATTTCGACTATGCGCTGGTGCAGATCGACGGTCAGGCCGTGATTCTGGCGAAAGATCTGGTTGAAAGCGTAATGCAGCGTATCGGCGTGACCGATTACACCATTCTCGGCACGGTAAAAGGTGCGGAGCTTGAGCTGCTGCGCTTTACCCATCCGTTTATGGGCTTCGACGTTCCGGCAATCCTCGGCGATCACGTTACCCTGGATGCCGGTACCGGTGCCGTTCACACCGCGCCTGGCCACGGCCCGGACGACTATGTGATCGGTCAGAAATACGGCCTGGAAACCGCTAACCCGGTTGGCCCGGACGGCACTTATCTGCCGGGCACTTATCCGACGCTGGATGGCGTGAACGTCTTCAAAGCGAACGACATCGTCGTTGCGCTGCTGCAAGAAAAAGGCGCGCTGCTGCACGTTGAGAAAATGCAGCACAGCTATCCGTGCTGCTGGCGTCACAAAACGCCGATCATCTTCCGCGCGACGCCGCAGTGGTTCGTCAGCATGGATCAGAAAGGTCTGCGTGCGCAGTCACTGAAAGAGATCAAAGGCGTGCAGTGGATCCCGGACTGGGGCCAGGCGCGTATCGAGTCGATGGTTGCTAACCGTCCTGACTGGTGTATCTCCCGTCAGCGCACCTGGGGTGTACCGATGTCACTGTTCGTGCACAAAGACACGGAAGAGCTGCATCCGCGTACCCTTGAACTGATGGAAGAAGTGGCAAAACGCGTTGAAGTCGATGGCATCCAGGCGTGGTGGGATCTCGATGCGAAAGAGATCCTCGGCGACGAAGCTGATCAGTACGTGAAAGTGCCGGACACATTGGATGTATGGTTTGACTCCGGATCTACCCACTCTTCTGTTGTTGACGTGCGTCCGGAGTTTGCCGGTCACGCAGCGGACATGTATCTGGAAGGTTCTGACCAACACCGCGGCTGGTTCATGTCTTCCCTAATGATCTCCACCGCGATGAAGGGTAAAGCGCCGTATCGTCAGGTACTGACCCACGGCTTTACCGTGGATGGTCAGGGCCGCAAGATGTCTAAATCCATCGGCAATACCGTTTCGCCGCAGGATGTGATGAACAAACTGGGCGCGGATATTCTGCGTCTGTGGGTGGCATCAACCGACTACACCGGTGAAATGGCCGTTTCTGACGAGATCCTGAAACGTGCTGCCGATAGCTATCGTCGTATCCGTAACACCGCGCGCTTCCTGCTGGCAAACCTGAACGGTTTTGATCCAGCAAAAGATATGGTGAAACCGGAAGAGATGGTGGTACTGGATCGCTGGGCCGTAGGTTGTGCGAAAGCGGCACAGGAAGACATCCTCAAGGCGTACGAAGCATACGATTTCCACGAAGTGGTACAGCGTCTGATGCGCTTCTGCTCCGTTGAGATGGGTTCCTTCTACCTCGACATCATCAAAGACCGTCAGTACACCGCCAAAGCGGACAGTGTGGCGCGTCGTAGCTGCCAGACTGCGCTATATCACATCGCAGAAGCGCTGGTGCGCTGGATGGCACCAATCCTCTCCTTCACCGCTGATGAAGTGTGGGGCTACCTGCCGGGCGAACGTGAAAAATACGTCTTCACCGGTGAGTGGTACGAAGGCCTGTTTGGCCTGGCAGACAGTGAAGCGATGAACGATGCGTTCTGGGACGAGCTGTTGAAAGTGCGTGGCGAAGTGAACAAAGTCATTGAGCAAGCGCGTGCCGACAAGAAAGTGGGTGGCTCGCTGGAAGCGGCAGTAACCTTGTATGCAGAACCGGAACTGTCGGCGAAACTGACCGCGCTGGGCGATGAATTACGATTTGTCCTGTTGACCTCCGGCGCTACCGTTGCAGACTATAACGACGCACCTGCTGATGCTCAGCAGAGCGAAGTACTCAAAGGGCTGAAAGTCGCGTTGAGTAAAGCCGAAGGTGAGAAGTGCCCACGCTGCTGGCACTACACCCAGGATGTCGGCAAGGTGGCGGAACACGCAGAAATCTGTGGCCGCTGTGTCAGCAACGTCGCCGGTGACGGTGAAAAACGTAAGTTTGCCCACCACCACCACCACCAC**TAA** |
| **His_6_-IF1 insert** |
| **Start** **Stop**  **ATG**CATCACCATCACCATCACGCGAAAGAAGATAATATTGAAATGCAAGGCACGGTCCTCGAAACGCTTCCGAACACGATGTTCCGGGTCGAGCTCGAAAACGGCCACGTCGTGACGGCGCATATCTCGGGCAAAATGCGCAAAAACTACATCCGGATCCTGACCGGCGACAAGGTCACCGTCGAACTCACGCCGTACGACCTCTCGAAAGGCCGCATCGTCTTCCGCTCGCGC**TAA** |
| **His_6_-IF3 insert** |
| **Start** **Stop**  **ATG**CATCACCATCACCATCACAAAGGCGGAAAACGAGTTCAAACGGCGCGCCCTAACCGTATCAATGGCGAAATTCGCGCCCAGGAAGTTCGCTTAACAGGTCTGGAAGGCGAGCAGCTTGGTATTGTGAGTCTGAGAGAAGCTCTGGAGAAAGCAGAAGAAGCCGGAGTAGACTTAGTCGAGATCAGCCCTAACGCCGAGCCGCCGGTTTGTCGTATAATGGATTACGGCAAATTCCTCTATGAAAAGAGCAAGTCTTCTAAGGAACAGAAGAAAAAGCAAAAAGTTATCCAGGTTAAGGAAATTAAATTCCGTCCTGGTACAGATGAAGGCGACTATCAGGTAAAACTCCGCAGCCTGATTCGCTTTCTCGAAGAGGGTGATAAAGCCAAAATCACGCTGCGTTTCCGCGGTCGTGAGATGGCGCACCAGCAAATCGGTATGGAAGTGCTTAATCGCGTGAAAGACGATTTGCAAGAACTGGCAGTGGTCGAATCCTTCCCAACGAAGATCGAAGGCCGCCAGATGATCATGGTGCTCGCTCCTAAGAAGAAACAG**TAA** |
| **His_6_-AlaRS insert** |
| **Start** **Stop**  **ATG**CACCACCACCACCACCACAGCAAGAGCACCGCTGAGATCCGTCAGGCGTTTCTCGACTTTTTCCATAGTAAGGGACATCAGGTAGTTGCCAGCAGCTCCCTGGTACCCCATAACGACCCAACTTTGTTGTTTACCAACGCCGGGATGAACCAGTTCAAGGATGTGTTCCTTGGGCTCGACAAGCGTAATTATTCCCGCGCTACCACTTCCCAACGCTGCGTGCGTGCGGGTGGTAAACACAACGACCTGGAAAACGTCGGTTACACCGCGCGTCACCATACCTTCTTCGAAATGCTGGGCAACTTCAGCTTCGGCGACTATTTCAAACACGATGCCATTCAGTTTGCATGGGAACTGCTGACCAGCGAAAAATGGTTTGCCCTGCCGAAAGAGCGTCTGTGGGTTACCGTCTATGAAAGCGACGACGAAGCCTACGAAATCTGGGAAAAAGAAGTAGGGATCCCGCGCGAACGTATTATTCGCATCGGCGATAACAAAGGTGCGCCATACGCATCTGACAACTTCTGGCAGATGGGTGACACTGGTCCGTGCGGCCCGTGCACCGAAATCTTCTACGATCACGGCGACCACATTTGGGGGGGCCCTCCGGGAAGCCCGGAAGAAGACGGCGACCGCTACATTGAGATCTGGAACATCGTCTTCATGCAGTTCAACCGCCAGGCCGATGGCACGATGGAACCGCTGCCGAAGCCGTCTGTAGATACCGGTATGGGTCTGGAGCGTATTGCTGCGGTGCTGCAACACGTTAACTCTAACTATGACATCGACCTGTTCCGCACGCTGATCCAGGCGGTAGCGAAAGTCACTGGCGCAACCGATCTGAGCAATAAATCGCTGCGCGTAATCGCTGACCACATTCGTTCTTGTGCGTTCCTGATCGCGGATGGCGTAATGCCGTCCAATGAAAACCGTGGTTATGTACTGCGTCGTATCATTCGTCGCGCAGTGCGTCACGGTAATATGCTCGGCGCGAAAGAAACCTTCTTCTACAAACTGGTTGGTCCGCTGATCGACGTTATGGGCTCTGCGGGTGAAGACCTGAAACGCCAGCAGGCGCAGGTTGAGCAGGTGCTGAAGACTGAAGAAGAGCAGTTTGCTCGTACTCTGGAGCGCGGTCTGGCGTTGCTGGATGAAGAGCTGGCAAAACTTTCTGGTGATACGCTGGATGGTGAAACTGCTTTCCGTCTGTACGACACCTATGGCTTCCCGGTTGACCTGACGGCTGATGTTTGTCGTGAGCGCAACATCAAAGTTGACGAAGCTGGTTTTGAAGCTGCAATGGAAGAGCAGCGTCGTCGCGCGCGCGAAGCCAGCGGCTTTGGTGCCGATTACAACGCAATGATCCGTGTTGACAGTGCATCTGAGTTTAAAGGCTATGACCATCTGGAACTGAACGGCAAAGTGACTGCGCTGTTTGTTGATGGTAAAGCGGTTGATGCCATCAATGCAGGCCAGGAAGCTGTGGTCGTGCTGGATCAAACGCCATTCTATGCGGAATCCGGCGGTCAGGTTGGCGATAAAGGCGAACTGAAAGGCGCTAACTTCTCCTTTGCGGTGGAAGATACGCAGAAATACGGCCAGGCGATTGGTCACATCGGTAAACTTGCTGCGGGTTCTCTGAAAGTGGGCGACGCGGTGCAGGCTGATGTTGATGAGGCTCGTCGCGCCCGTATTCGTCTGAATCACTCCGCAACGCACCTGATGCACGCTGCGCTGCGCCAGGTTCTGGGTACTCATGTATCGCAGAAAGGTTCACTGGTTAACGACAAGGTGCTGCGCTTCGACTTCTCACACAACGAAGCGATGAAACCAGAAGAGATTCGTGCGGTCGAAGACCTGGTGAACACACAGATTCGTCGCAATTTGCCGATCGAAACCAACATCATGGATCTCGAAGCGGCGAAAGCGAAAGGTGCGATGGCGCTGTTCGGCGAGAAGTATGATGAGCGCGTACGCGTGCTGAGCATGGGCGATTTCTCTACCGAGTTGTGTGGCGGTACTCACGCCAGCCGCACTGGTGATATTGGTCTGTTCCGCATCATCTCTGAATCGGGTACTGCTGCCGGCGTTCGTCGTATCGAAGCGGTAACCGGAGAAGGTGCTATCGCCACCGTTCATGCAGACAGCGATCGCTTAAGCGAAGTCGCGCATCTGCTGAAAGGCGATAGCAATAATCTGGCTGATAAAGTGCGCTCAGTACTGGAACGTACGCGTCAGCTGGAAAAAGAGTTACAACAGCTTAAAGAACAAGCTGCCGCACAGGAGAGCGCAAATCTTTCCAGTAAGGCAATCGATGTTAATGGTGTTAAGCTGTTGGTTAGCGAGCTTAGCGGTGTTGAGCCGAAAATGTTGCGTACCATGGTTGACGATTTAAAAAATCAGCTGGGGTCGACAATTATCGTGCTGGCAACGGTAGTCGAAGGTAAGGTTTCTCTGATTGCAGGCGTATCTAAGGACGTCACAGATCGTGTGAAAGCAGGGGAACTGATTGGTATGGTCGCTCAGCAGGTGGGCGGCAAGGGTGGTGGACGTCCTGACATGGCGCAAGCCGGTGGTACGGATGCTGCGGCCTTACCGGCAGCGTTAGCCAGTGTGAAAGGCTGGGTCAGCGCGAAATTGCAA**TAA** |
| **His_6_-AsnRS insert** |
| **Start** **Stop**  **ATG**CACCACCACCACCACCACAGCGTTGTGCCTGTAGCCGACGTACTCCAGGGCCGTGTAGCCGTTGACAGCGAAGTCACCGTGCGCGGATGGGTACGTACCCGCCGAGATTCAAAAGCTGGCATCTCCTTCCTCGCCGTTTATGACGGTTCCTGCTTTGATCCTGTACAGGCTGTCATCAATAATTCTCTGCCCAATTACAATGAAGACGTCCTGCGTCTGACCACCGGCTGCTCGGTCATTGTGACGGGTAAAGTCGTGGCGTCGCCGGGCCAGGGGCAACAATTTGAAATCCAGGCCAGCAAGGTTGAAGTTGCTGGTTGGGTTGAAGATCCAGACACTTACCCGATGGCGGCAAAACGCCACAGCATTGAGTATCTGCGTGAAGTCGCTCACCTGCGTCCGCGCACAAACCTGATTGGTGCCGTCGCGCGCGTTCGCCATACGCTGGCGCAGGCGCTGCATCGCTTCTTTAACGAGCAGGGATTCTTCTGGGTTTCAACGCCACTGATTACCGCATCTGATACCGAAGGTGCAGGCGAAATGTTCCGCGTTTCTACGCTGGATCTGGAAAACCTGCCGCGTAACGATCAGGGCAAAGTGGATTTCGACAAAGACTTCTTTGGTAAAGAGTCTTTCCTGACCGTATCTGGCCAGTTGAACGGCGAAACCTACGCTTGCGCATTGTCCAAAATCTATACCTTCGGCCCGACTTTCCGTGCTGAAAACTCCAACACCAGCCGTCACCTGGCGGAGTTCTGGATGCTGGAGCCGGAAGTGGCGTTTGCTAACCTGAACGATATTGCGGGTCTGGCTGAAGCCATGCTGAAATATGTCTTCAAAGCGGTTCTCGAAGAACGCGCTGACGACATGAAGTTCTTCGCTGAACGCGTAGATAAAGATGCCGTTTCACGTCTGGAACGCTTCATTGAAGCCGATTTTGCGCAGGTGGATTATACCGACGCAGTGACCATTCTCGAAAACTGCGGCAGGAAGTTTGAAAACCCGGTTTACTGGGGAGTCGATCTCTCTTCTGAGCATGAGCGTTATCTGGCGGAAGAACACTTTAAAGCACCGGTAGTGGTTAAAAACTATCCGAAAGATATTAAAGCGTTCTATATGCGCCTTAACGAAGACGGTAAAACCGTTGCGGCTATGGACGTTCTGGCTCCGGGCATCGGTGAGATCATTGGTGGCTCCCAGCGTGAAGAACGTCTGGACGTGCTGGACGAGCGTATGCTGGAAATGGGCCTGAATAAAGAAGATTACTGGTGGTATCGCGATCTGCGTCGCTACGGTACTGTTCCGCATTCAGGTTTCGGTCTTGGTTTTGAACGTCTGATTGCTTACGTAACTGGCGTGCAAAACGTACGTGATGTGATTCCGTTCCCACGTACTCCGCGTAACGCCAGCTTC**TAA** |
| **His_6_-PheRSα + PheRSβ insert** |
| **Start** **Stop** PheRSα PheRSβ  **ATG**CACCACCACCACCACTCACATCTCGCAGAACTGGTTGCCAGTGCGAAGGCGGCCATTAGCCAGGCGTCAGATGTTGCCGCGTTAGATAATGTGCGCGTCGAATATTTGGGTAAAAAAGGGCACTTAACCCTTCAGATGACGACCCTGCGTGAGCTGCCGCCAGAAGAGCGTCCGGCAGCTGGTGCGGTTATCAACGAAGCGAAAGAGCAGGTTCAGCAGGCGCTGAATGCGCGTAAAGCGGAACTGGAAAGCGCTGCACTGAATGCGCGTCTGGCGGCGGAAACGATTGATGTCTCTCTGCCAGGTCGTCGCATTGAAAACGGCGGTCTGCATCCGGTTACCCGTACCATCGACCGTATCGAAAGTTTCTTCGGTGAGCTTGGCTTTACCGTGGCAACCGGGCCGGAAATCGAAGACGATTATCATAACTTCGATGCTCTGAACATTCCTGGTCACCACCCGGCGCGCGCTGACCACGACACTTTCTGGTTTGACACTACCCGCCTGCTGCGTACCCAGACCTCTGGCGTACAGATCCGCACCATGAAAGCCCAGCAGCCACCGATTCGTATCATCGCGCCTGGCCGTGTTTATCGTAACGACTACGACCAGACTCACACGCCGATGTTCCATCAGATGGAAGGTCTGATTGTTGATACCAACATCAGCTTTACCAACCTGAAAGGCACGCTGCACGACTTCCTGCGTAACTTCTTTGAGGAAGATTTGCAGATTCGCTTCCGTCCTTCCTACTTCCCGTTTACCGAACCTTCTGCGGAAGTGGACGTCATGGGTAAAAACGGTAAATGGCTGGAAGTGCTGGGCTGCGGGATGGTGCATCCGAACGTGTTGCGTAACGTTGGCATCGACCCGGAAGTTTACTCTGGTTTCGCCTTCGGGATGGGGATGGAGCGTCTGACTATGTTGCGTTACGGCGTCACCGACCTGCGTTCATTCTTCGAAAACGATCTGCGTTTCCTCAAACAGTTTAAA**TAA**GGCAGGAATAGATTATGAAGTTCAGTGAACTGTGGTTACGCGAATGGGTGAACCCGGCGATTGATAGCGATGCGCTGGCAAATCAAATCACT**ATG**GCGGGCCTGGAAGTTGACGGTGTAGAACCGGTTGCCGGCAGCTTCCACGGCGTGGTCGTTGGTGAAGTGGTTGAGTGTGCGCAGCATCCGAACGCTGACAAACTGCGTGTGACAAAAGTGAATGTCGGCGGCGATCGCCTGCTGGACATCGTCTGCGGTGCGCCAAACTGCCGTCAGGGCCTGCGTGTAGCGGTAGCGACCATTGGTGCTGTTCTGCCGGGTGATTTCAAAATTAAAGCGGCGAAACTGCGTGGCGAACCGTCTGAAGGGATGCTGTGCTCCTTCTCTGAACTGGGCATTTCTGACGATCACAGCGGCATTATCGAACTGCCTGCGGATGCGCCGATTGGCACCGATATCCGTGAATACCTGAAACTTGATGACAACACCATCGAAATCAGCGTGACGCCAAACCGTGCCGACTGCTTAGGCATCATTGGTGTTGCGCGTGACGTTGCCGTGCTGAACCAGCTGCCGCTGGTTCAACCGGAAATCGTTCCGGTTGGTGCGACCATCGACGACACGCTGCCGATTACAGTCGAAGCGCCGGAAGCCTGCCCGCGTTATCTTGGCCGTGTGGTAAAAGGCATTAACGTTAAAGCGCCAACTCCGCTGTGGATGAAAGAAAAACTGCGTCGTTGCGGGATCCGTTCTATCGATGCAGTTGTTGACGTCACCAACTATGTGCTGCTCGAACTGGGCCAGCCGATGCACGCTTTCGATAAAGATCGCATTGAAGGCGGCATTGTGGTGCGGATGGCGAAAGAGGGCGAAACGCTGGTGCTGCTCGACGGTACTGAAGCGAAGCTGAATGCTGACACTCTGGTCATCGCCGACCACAACAAGGCGCTGGCGATGGGCGGCATCTTCGGTGGCGAACACTCTGGCGTGAATGACGAAACACAAAACGTGCTGCTGGAATGCGCGTTCTTTAGCCCGCTGTCTATCACCGGTCGTGCTCGTCGTCATGGCCTGCATACCGATGCGTCTCACCGTTATGAGCGTGGCGTTGATCCGGCACTGCAACACAAAGCGATGGAACGTGCGACCCGTCTGCTGATCGACATCTGCGGTGGTGAGGCTGGCCCGGTAATTGATATCACCAACGAAGCAACGCTGCCGAAGCGTGCAACCATCACTCTACGTCGTAGCAAACTGGATCGCCTGATCGGCCATCATATTGCGGATGAGCAGGTAACTGACATTCTGCGTCGTCTCGGCTGCGAAGTGACCGAAGGCAAAGACGAATGGCAGGCAGTTGCGCCGAGCTGGCGTTTCGATATGGAGATTGAAGAAGATCTGGTTGAAGAAGTCGCGCGTGTTTACGGCTACAACAACATCCCGGATGAGCCGGTACAGGCAAGCCTGATTATGGGTACTCACCGTGAAGCTGACCTGTCGCTCAAGCGCGTGAAAACGCTGCTCAACGACAAAGGCTATCAGGAAGTGATCACCTACAGCTTCGTTGATCCGAAAGTGCAGCAGATGATCCATCCAGGCGTTGAAGCCTTACTGCTGCCAAGCCCGATCTCTGTTGAAATGTCAGCAATGCGTCTTTCTCTGTGGACTGGCCTGCTGGCAACCGTGGTGTACAACCAGAACCGTCAGCAGAACCGTGTGCGCATTTTCGAAAGCGGTCTGCGTTTCGTACCAGATACTCAGGCACCGTTGGGCATTCGTCAGGATCTGATGTTAGCCGGTGTGATTTGCGGTAACCGTTACGAAGAGCACTGGAACCTGGCAAAAGAGACCGTTGATTTCTATGATTTGAAAGGCGATCTTGAATCCGTTCTCGACCTGACCGGTAAACTGAATGAGGTTGAGTTCCGTGCAGAAGCGAATCCGGCACTGCATCCGGGGCAATCCGCAGCGATTTATCTGAAAGGTGAACGTATTGGTTTTGTTGGGGTTGTTCATCCTGAACTGGAACGTAAACTGGATCTTAACGGTCGCACTCTGGTGTTCGAACTGGAGTGGAACAAGCTCGCAGACCGCGTGGTGCCTCAGGCGCGCGAGATTTCTCGCTTCCCGGCGAACCGTCGTGACATCGCGGTGGTGGTCGCAGAAAACGTTCCCGCAGCGGATATTTTATCCGAATGTAAGAAAGTTGGCGTAAATCAGGTAGTTGGCGTAAACTTATTTGACGTGTACCGCGGTAAGGGTGTTGCGGAGGGGTATAAGAGCCTCGCCATAAGCCTGATCCTGCAAGATACCAGCCGTACACTCGAAGAAGAGGAGATTGCCGCTACCGTCGCCAAATGTGTAGAGGCATTAAAAGAGCGATTCCAGGCATCATTGAGGGAT**TAA** |
